# TGFβ selects for pro‐stemness over pro‐invasive phenotypes during cancer cell epithelial–mesenchymal transition

**DOI:** 10.1002/1878-0261.13215

**Published:** 2022-04-10

**Authors:** Yutaro Tsubakihara, Yae Ohata, Yukari Okita, Shady Younis, Jens Eriksson, Mikael E. Sellin, Jiang Ren, Peter ten Dijke, Kohei Miyazono, Atsuhiko Hikita, Takeshi Imamura, Mitsuyasu Kato, Carl‐Henrik Heldin, Aristidis Moustakas

**Affiliations:** ^1^ Department of Medical Biochemistry and Microbiology Science for Life Laboratory Biomedical Center Uppsala University Sweden; ^2^ Department of Experimental Pathology and Transborder Medical Research Center Faculty of Medicine University of Tsukuba Japan; ^3^ Division of Immunology and Rheumatology Department of Medicine Stanford University Stanford CA USA; ^4^ Department of Cell and Chemical Biology Oncode Institute Leiden University Medical Center Leiden The Netherlands; ^5^ Department of Molecular Pathology Graduate School of Medicine The University of Tokyo Bunkyo‐ku Japan; ^6^ Divison of Tissue Engineering The University of Tokyo Hospital Bunkyo‐ku Japan; ^7^ Department of Molecular Medicine for Pathogenesis Graduate School of Medicine Ehime University Toon Japan

**Keywords:** cancer stem cell, E‐cadherin, epithelial‐mesenchymal transition, invasion, metastasis, transforming growth factor β

## Abstract

Transforming growth factor β (TGFβ) induces epithelial–mesenchymal transition (EMT), which correlates with stemness and invasiveness. Mesenchymal–epithelial transition (MET) is induced by TGFβ withdrawal and correlates with metastatic colonization. Whether TGFβ promotes stemness and invasiveness simultaneously *via* EMT remains unclear. We established a breast cancer cell model expressing red fluorescent protein (RFP) under the *E‐cadherin* promoter. In 2D cultures, TGFβ induced EMT, generating RFP^low^ cells with a mesenchymal transcriptome, and regained RFP, with an epithelial transcriptome, after MET induced by TGFβ withdrawal. RFP^low^ cells generated robust mammospheres, with epithelio‐mesenchymal cell surface features. Mammospheres that were forced to adhere generated migratory cells, devoid of RFP, a phenotype which was inhibited by a TGFβ receptor kinase inhibitor. Further stimulation of RFP^low^ mammospheres with TGFβ suppressed the generation of motile cells, but enhanced mammosphere growth. Accordingly, mammary fat‐pad‐transplanted mammospheres, in the absence of exogenous TGFβ treatment, established lung metastases with evident MET (RFP^high^ cells). In contrast, TGFβ‐treated mammospheres revealed high tumour‐initiating capacity, but limited metastatic potential. Thus, the biological context of partial EMT and MET allows TGFβ to differentiate between pro‐stemness and pro‐invasive phenotypes.

## Graphical abstract

Whether TGFβ promotes stemness and invasiveness simultaneously *via* epithelial‐mesenchymal transition (EMT) still remains unclear. Using a breast cancer cell model expressing red fluorescent protein under the E‐cadherin promoter, we demonstrate that the biological context of partial mesenchymal–epithelial transition (pMET) enriches mammospheres with invasive and pro‐metastatic potential. Sustained partial EMT under TGFβ enriches mammospheres with a pro‐stemness phenotype with higher tumour‐initiating potential.

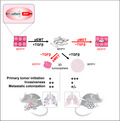

**Abbreviations**
BMP: bone morphogenetic protein
CMV: cytomegalovirus
EGFP: enhanced green fluorescent protein
ELDA: extreme limiting dilution assay
EMT: epithelial‐mesenchymal transition
GO: gene ontology
GSEA: gene set enrichment analysis
MET: mesenchymal‐epithelial transition
RFP: red fluorescent protein
TF: transcription factor
TGFβ: transforming growth factor β

## 1 Introduction

Epithelial–mesenchymal transition (EMT) is activated at distinct developmental stages and anatomical sites, e.g., during primitive streak, neural crest and heart cushion formation [1]. *Via* EMT, epithelia disseminate migratory cells that colonize new embryonic locales and differentiate into new tissues [1]. Strong evidence supports a role for EMT also in cancer cell invasiveness, chemoresistance and metastasis [2]. Accordingly, EMT alters the differentiation of epithelial cells causing the suppression of tight junctional components, of adherens junctional proteins, such as E‐cadherin, and induction of N‐cadherin [1, 3]. The latter marks an adaptation of the epithelial apicobasal polarity to the posterior‐interior polarity of mesenchymal cells. Moreover, the exchange of integrin receptors remodels hemidesmosome‐mediated basal membrane adhesion to focal adhesion‐mediated motility. Changes in these adhesion complexes are supported by the reorganization of intermediate filaments, i.e., lowering expression of specific cytokeratins by increasing levels of vimentin and cytokeratin‐14, and extensive remodeling of actin microfilaments and secreted extracellular proteins, including matrix proteins and cytokines [1, 3].

The cytokine transforming growth factor β (TGFβ) and its family members (e.g., nodal) drive EMT during development and cancer progression [4, 5, 6]. TGFβ thus generates cells of a mesenchymal phenotype, including cancer‐associated fibroblasts, and promotes invasive spread and metastatic potential in several tumour types [4, 6, 7]. TGF‐β signaling is transmitted *via* the TGFβ type II and type I receptor kinases; downstream Smad, mitogen‐activated protein kinases, phospholipid kinases and small GTPases, mediate transcriptional induction and post‐transcriptional stabilization of a cohort of transcription factors that initiate the EMT (EMT‐TFs), such as Snail, Slug, Zeb1, Zeb2, Twist1, Twist2 and Prrx1, and associated chromatin factors (e.g. Hmga2); Snail, Zeb1, Zeb2 and Hmga2 also form transcriptional complexes with Smads [1, 3, 8].

Similar to other changes in cell differentiation, EMT proceeds with slow kinetics *in vitro* and *in vivo* and is reversible, leading to mesenchymal‐epithelial transition (MET) [1]. Slow progression of EMT, or possibly EMT followed by inefficient MET, the latter not being documented experimentally, can lead to intermediary cellular phenotypes, often called hybrid epithelial/mesenchymal phenotype or partial EMT phenotype, generating epithelio‐mesenchymal cells with unique biological properties, as demonstrated under given biological contexts, and experimentally defined by the presence of specific cell surface proteins [2]. Epithelio‐mesenchymal phenotypes have been supported by mathematical models of TGFβ‐mediated EMT dynamics in culture [9, 10, 11, 12]. Accordingly, distinct epithelial to epithelio‐mesenchymal transition, due to the action of Snail and its negative regulator microRNA *miR34*, further progresses towards epithelio‐mesenchymal to mesenchymal transition, *via* the action of ZEB1 and its negative regulator *miR200* [9, 11, 12, 13, 14]. Alternatively, sequential induction of Snail and Prrx1 by TGFβ, followed by Prrx1‐mediated induction of *miR15*, which downregulates Snail, generates an equivalent binary regulatory network relevant to normal developmental and breast or lung cancer EMT [15]. EMT may also depend on a more complex network of transcription factor‐miRNA interactions [16]. Thus, single cell RNAseq analyses of TGFβ‐induced EMT have suggested multiple sequential regulatory cascades that include the Ras and Notch pathways that crosstalk with TGFβ, possibly producing a continuum of subtle biological alterations that cumulatively establish the EMT [17, 18]. A clear role of intermediate EMT stages in metastasis has been demonstrated in experimental breast and skin cancer models, proposing that cell invasiveness strongly associates with the epithelio‐mesenchymal phenotype [19, 20, 21, 22].

The fact that TGFβ induces all facets of EMT, poses the important question whether distinct phenotypes that are associated with the EMT, such as cancer cell stemness and motility, are selected during different biological contexts. To address this question, we established a fluorescent breast cancer cell model and analyzed the effect of TGFβ on epithelial and mesenchymal cell behaviour in 2D and 3D cultures, as well as on tumour growth and metastasis *in vivo*.

## 2 Materials and methods

### 2.1 2D culture

Mouse breast cancer Py2T cells from a breast tumour of an MMTV‐PyMT transgenic mouse [23] were kindly donated by Dr. Gerhard Christofori (University of Basel, Basel, Switzerland). Py2T cells were cultured in Dulbecco’s modified Eagle’s medium (DMEM, Sigma‐Aldrich AB, Stockholm, Sweden), supplemented with 10% fetal bovine serum (FBS; Biowest, Almeco A/S, Esbjerg, Denmark), 100 U·mL^−1^ penicillin and 100 μg·mL^−1^ streptomycin (Sigma‐Aldrich AB, Stockholm, Sweden). The human male lung adenocarcinoma A549 cells and human female breast cancer MDA‐MB‐231 cells were from the American Type Culture Collection, Manassas, VA, USA. A549 and MDA‐MB‐231 cells were cultured in Dulbecco’s modified Eagle’s medium (DMEM, Sigma‐Aldrich AB, Stockholm, Sweden), supplemented with 10% fetal bovine serum (FBS; Biowest, Almeco A/S, Esbjerg, Denmark), 100 U·mL^−1^ penicillin and 100 μg·mL^−1^ streptomycin (Sigma‐Aldrich AB, Stockholm, Sweden). Mouse mammary gland epithelial EpH4 cells transformed with mutant H‐Ras [24] were kindly donated by the late Dr. Hartmut Beug (Institute of Molecular Pathology, Vienna, Austria), and were maintained in DMEM supplemented with 4% FBS, 2 mm l‐glutamine, 20 mm HEPES, 100 U·mL^−1^ penicillin and 100 μg·mL^−1^ streptomycin. Human transformed female breast epithelial MCF10AT1k.cl2 (MII) and MCF10ACA1h (MIII) cells [25] (kindly donated by Dr. Robert J. Pauley, Barbara Ann Karmanos Cancer Institute, Detroit, MI, USA), human female mammary epithelial cells (HMEC) (from Thermo Fisher Scientific/Lonza/Clonetics, and HMEC stably immortalized by the large T‐antigen (HMLE) [26] kindly donated by Dr. Robert A. Weinberg (Whitehead Institute for Biomedical Research and Massachusetts Institute of Technology, Cambridge, MA, USA)) were maintained in DMEM/F12 (Sigma‐Aldrich AB, Stockholm, Sweden) supplemented with 5% FBS, 2 mm l‐glutamine (Sigma‐Aldrich AB, Stockholm, Sweden), 20 ng·mL^−1^ epidermal growth factor (PeproTech EC Ltd, London, UK), 100 ng·mL^−1^ cholera toxin (Sigma‐Aldrich AB, Stockholm, Sweden), 0.5 µg·mL^−1^ hydrocortisone (Sigma‐Aldrich AB, Stockholm, Sweden), 10 µg·mL^−1^ insulin (Thermo Fisher Scientific, Stockholm, Sweden), 100 U·mL^−1^ penicillin, and 100 µg·mL^−1^ streptomycin. All cell lines were cultured at 37 °C in a 5% CO_2_ atmosphere, were free of mycoplasma (tested every 4 months), and human cell lines were authenticated using PCR‐single‐locus‐technology (Eurofins, Uppsala, Sweden). For additional features of the cell lines used, we refer the reader to the Tables S1 and S2.

### 2.2 3D culture

*E‐cadherin*‐RFP/Py2T cells were stimulated with TGFβ1 or BMP7 in 2D culture condition for 7 days; after trypsinization, 1 × 10^6^ cells were recovered and cultured in ultra‐low attachment flasks (Corning, NY, USA) with/without TGFβ1 or BMP7 for 5 days. Mammosphere size was calculated by the image j software (National Institutes of Health, Bethesda, MD, USA).

### 2.3 Reagents and treatments

The cells were treated with recombinant human TGFβ1 (5 ng·mL^−1^, PeproTech EC Ltd, London, UK) and recombinant human BMP7 (100 ng·mL^−1^, a gift from K. Sampath, Sanofi‐Gelisa Research Center, Framingham, MA, USA) for the time periods indicated in the Figures. The LY2157299 TβRI kinase inhibitor (Galunisertib; Sigma‐Aldrich AB, Stockholm, Sweden) was administered to cells at a final concentration of 2.5 μm. Anti‐cancer drugs, 5´‐fluoro‐uracil (5‐FU, F6627, Sigma‐Aldrich AB, Stockholm, Sweden), cisplatin (232120, Sigma‐Aldrich AB, Stockholm, Sweden), cyclophosphamide (Toronto Research Chemicals, North York, Canada), doxorubicin (D1515, Sigma‐Aldrich AB, Stockholm, Sweden) and taxol (T7402, Sigma‐Aldrich AB, Stockholm, Sweden) were used for chemoresistance experiments. Dimethyl‐sulfoxide (DMSO) served as a vehicle for all chemicals and additional details are presented in Table S3.

### 2.4 Mouse transplantation and tumour volume calculation

*E‐cadherin*‐RFP/Py2T cells (1 × 10^5^) were mixed in a 1 : 1 ratio of phosphate buffered saline (PBS) and Matrigel Growth Factor Reduced Basement Membrane Matrix (356230, Corning, NY, USA) and orthotopically injected into two mammary fat pads (2 sites per mouse) of 6‐week old female Balb/c nu/nu (Nude) mice (CLEA Japan, Tokyo, Japan). Tumour incidence was analyzed every 2 days for the presence of primary tumour in mammary fat pads. Six weeks after transplantation, mice were sacrificed, primary tumours and whole lungs were dissected and processed for immunohistochemistry and analyzed for metastasis. Primary tumours were measured to calculate their major and minor diameters. Tumour volume (cm^3^) was calculated by the following formula: [(major dimension, cm)×(minor dimension, cm)^2^]/2. Mice were maintained under specific pathogen‐free conditions with 14 h‐light/10 h‐dark cycles at 23.5 ± 2.5 °C and 52.5 ± 12.5% relative humidity. Mice were free to access their food and water. All mouse experiments were performed with the approval of the Animal Ethics Committee of the University of Tsukuba (Ibaraki, Japan; approval number: 20‐207).

### 2.5 Zebrafish extravasation and tail invasion assay

Zebrafish experiments were conducted according to international guidelines and were approved by the local Institutional Committee for Animal Welfare (Dier Ethische Commissie (DEC)) of Leiden University Medical Center, The Netherlands. The zebrafish xenograft model was established as described before [27, 28]. Briefly, zebrafish embryos were maintained at 28 °C. At 48 h post‐fertilization, approximately 400 *E‐cadherin*‐RFP/Py2T or *CMV*‐RFP/Py2T cells were injected into the duct of Cuvier of transgenic zebrafish embryos (*fli1*: enhanced green fluorescent protein (EGFP)), whose vasculature is marked in green. After verification by microscopy, only correctly injected and viable zebrafish were retained and maintained at 34 °C, as a compromise for the viability of both the fish and the mammalian cell lines. Five days post‐implantation (dpi), the cells that extravasated from the circulation at the posterior part of the zebrafish were imaged and counted under a confocal microscope (SP5 STED, Leica Microsystems, Wetzlar, Germany). All experiments were repeated at least two times independently, and representative experiments are shown.

### 2.6 Plasmids

The mouse *E‐cadherin*, mouse *Esrp2* or human *miR‐200s* promoter‐tdTomato (RFP) plasmids were generated by subcloning the *E‐cadherin* [29], *Esrp2* [30] or *miR200s* [13] promoter from matched luciferase reporter plasmids. The subcloned promoters were independently ligated with a tdTomato plasmid lacking the *EF1α* promoter [31]. *E‐cadherin* promoter‐luc, *Esrp2* promoter‐luc [30], pcDNA3‐Snail‐HA [32], ‐Slug‐HA [32], ‐Flag‐ZEB1 [33], ‐Flag‐ZEB2 [33] and ‐Flag‐Twist1 plasmids were kindly provided by Dr. Masao Saitoh (Yamanashi University, Japan). The human *miR‐200s* promoter‐luc plasmid was kindly provided by Dr. Gregory J. Goodall [13] (University of South Australia, Adelaide, Australia). The CAGA_9_‐luc reporter plasmid was previously described [34]. All recombinant DNA plasmids used are listed in Table S4. The cloning primer sequences used to generate the plasmids are listed in Table S5.

### 2.7 Transfections with plasmids and generation of the EMTimage

Transient transfections of Py2T, A549, MCF10A MII or EpRas cells with plasmids were performed using Lipofectamine 3000 (Invitrogen, Thermo Fisher Scientific, Stockholm, Sweden). For the generation of stable mouse *E‐cadherin* promoter‐RFP, mouse *Esrp2* promoter‐RFP or human *miR‐200s* promoter‐RFP overexpressing cells, transfections were as described above, and 1.0 mg·mL^−1^ geneticin (Thermo Fisher Scientific, Stockholm, Sweden) was added to the cells 48 h after transfection, followed by culture in the presence of geneticin for 2 weeks. The top 10% RFP‐highly expressing Py2T cell population was sorted from the pool of transfected cells by flow cytometry; after repeating the sorting twice, the sorted cells were grown in culture medium with geneticin.

### 2.8 Transient transfections and luciferase assays

Py2T, A549, EpRas or MCF10A MII cells transiently transfected with the epithelial gene promoter reporter constructs (*E‐cadherin*‐luc, *Esrp2*‐luc, *miR‐200s*‐luc and *CAGA_9_*‐luc) were used for luciferase assays. *TK‐Renilla* luciferase reporter plasmid (pGL4.74, Promega, Madison, Wisconsin, USA) was co‐transfected with the transiently transfected promoter reporter plasmids for normalization of the firefly luciferase measurements. Luciferase reporter assays were performed using the Firefly and Renilla Dual Luciferase Assay kit (Biotium, Fremont CA, USA, Cat# BTIU30003‐2). Relative normalized luciferase activity from triplicate determinations derived average values with standard deviations and is presented in the graphs. Each experiment was repeated at least three times.

### 2.9 cDNA synthesis and real‐time PCR

Total RNA was extracted using TRIzol (Thermo Fisher Scientific, Stockholm, Sweden). The concentration of RNA was measured using a NanoDrop 2000 instrument (Thermo Fisher Scientific, Stockholm, Sweden), and 1 μg of RNA was used for reverse transcription using the iScript cDNA synthesis kit (Bio‐Rad Laboratories AB, Solna, Sweden, Cat# 170‐8891), according to the protocol by the manufacturer. Real‐time qPCR was performed on a Bio‐Rad CFX96 cycler (Bio‐Rad Laboratories AB, Solna, Sweden) using the qPCRBIO SyGreen 2×Master Mix (PCR Biosystems, London, UK, Cat# 22‐PB20.13‐50). The expression levels of target genes were normalized to the levels of the reference gene *GAPDH*/*Gapdh* and relative normalized expression was calculated based on the standard curve method. The results were plotted in graphs as average values of relative normalized expression with standard deviations of at least three biological experiments. The oligonucleotide sequences used for RT‐qPCR are listed in Table S5.

### 2.10 Immunoblotting

Total proteins were extracted using lysis buffer (20 mm Tris‐HCl, pH 7.5, 1% Nonidet P‐40, 150 mm NaCl, 10 mm EDTA), supplemented with complete protease inhibitor mixture (Roche Diagnostics Scandinavia AB, Bromma, Sweden). The lysates were cleared by centrifugation at 15 000 × ***g*** for 15 min. The supernatant was transferred to new tubes and protein concentration was measured with the Bradford assay (Protein Assay Dye Reagent Concentrate, Bio‐Rad Laboratories AB, Solna, Sweden, Cat# 5000006). Next, one fifth volume of 5× sample buffer (150 mm Tris‐HCl, pH 6.8, 15% SDS, 25% glycerol, 0.02% bromophenol blue, 12.5% β‐mercaptoethanol) and water was added to the lysates, which were then boiled at 95 °C for 5 min and subjected to SDS/PAGE. Equal amounts of protein (40 μg) were loaded to each polyacrylamide gel. The resolved proteins were transferred to a nitrocellulose filter using a wet transfer unit (Bio‐Rad Laboratories AB, Solna, Sweden). After the transfer, blocking was done by 5% BSA or 5% skim milk, and then the filters were washed by Tris buffered saline‐Tween (19 mm Tris‐base, pH 7.4, 137 mm NaCl, 2.7 mm KCl, 0.1% v/v Tween‐20) and incubated with primary antibodies and horseradish peroxidase‐conjugated secondary antibodies, and then, enhanced chemiluminescence assays were performed using the Millipore kit (Merck/Millipore, Stockholm, Sweden, Cat# WBKLS0500). The antibodies used and their dilution factors are listed in Table S6. All original immunoblots are presented in the supplemental information section.

### 2.11 Immunofluorescence

*E‐cadherin*‐RFP/Py2T cells were fixed in 3.7% (w/v) formaldehyde stabilized with 10% (v/v) methanol for 10 min at room temperature. The cells were permeabilized with 0.1% v/v Triton X‐100 for 10 min at room temperature and blocked in PBS supplemented with 5% FBS for 1 h at room temperature, followed by incubation with primary antibodies overnight at 4 °C. The next day, the samples were incubated with Alexa Fluor‐488‐labelled secondary antibody (Invitrogen, Thermo Fisher Scientific, Stockholm, Sweden) at a dilution of 1 : 300 in 5% v/v FBS/PBS for 1 h at 4 °C. Phalloidin staining was also performed using tetramethyl‐rhodamine‐isothiocyanate‐conjugated phalloidin (diluted 1 : 1000; Sigma‐Aldrich AB, Stockholm, Sweden) in PBS supplemented with 5% FBS for 30 min at 4 °C. The samples were then mounted using ProLong Gold Antifade Mountant with 4’, 6‐diamidino‐2‐phenylindole (DAPI, Invitrogen, Thermo Fisher Scientific, Stockholm, Sweden, Cat# P36931) and examined on a Zeiss Axioplan 2 fluorescence microscope or a Zeiss LSM700 confocal microscope with the Zeiss 40× or 20× objective lens (Carl Zeiss AB, Stockholm, Sweden). The antibodies used and their dilution factors are listed in Table S6.

### 2.12 RNAseq transcriptome analysis

Total RNA from untreated *E‐cadherin* promoter‐RFP/Py2T cells (control), cells treated with TGFβ1 for 7 days (EMT) and cells treated with TGFβ1 for 7 days followed by withdrawal of TGFβ1 for 7 days (MET) was isolated using the PureLink RNA Mini kit (Thermo Fisher Scientific, Stockholm, Sweden, Cat # A21206). Strand‐specific mRNA sequencing libraries were generated using SENSE library prep kit (Lexogen GmbH, Vienna, Austria, Cat# 001.24) following the manufacturer's instructions. Briefly, 1 µg of total RNA was poly‐A selected using magnetic beads. The Illumina‐compatible linker sequences were introduced to the RNA by random primer hybridization. The amplified libraries were size‐selected for average insert size around 350 bp of the polyadenylated RNA. The libraries were sequenced as 50 bp single reads using an Illumina HiSeq instrument at SciLifeLab, Uppsala.

Sequence reads were mapped to the reference mouse genome (mm10) using STAR 2.5.1b with default parameters [35, 36]. HTSeq‐0.6.1 (Python Package) was used to generate read counts and edgeR (Bioconductor package) [37] to analyze differentially expressed (DE) genes using gene models for mm10 downloaded from UCSC (www.genome.ucsc.edu). The abundance of gene expression was calculated as count‐per‐million (CPM) reads. Genes with less than three CPM in at least three samples were filtered out. The filtered libraries were normalized using the trimmed mean of m‐values (TMM) normalization method. Adjusted *P*‐values (padj) for multiple testing, using Benjamini‐Hochberg to estimate the false discovery rate (FDR), were calculated for final estimation of DE significance.

For gene ontology analysis, the DE genes were analyzed using the Clusterprofiler R package [38]. All expressed genes were used as background, and the Biological Process and KEGG pathway tables were used to identify enriched GO terms. The gene set enrichment analysis (GSEA) was performed using the fgsea R package [39]. The genes were ranked based on the fold‐change and the datasets were downloaded from the GSEA website (https://www.gsea‐msigdb.org/gsea/downloads.jsp). All RNAseq primary data and protocols have been submitted to Arrayexpress, EBI, UK under accession number E‐MTAB‐9750. Detailed RNAseq analysis files are presented in Tables [Link], [Link], [Link], [Link], [Link].

### 2.13 Invasion assays

*E‐cadherin*‐RFP/Py2T cells were stimulated with TGFβ1 in 2D condition for 12 days. Subsequently, the cells were seeded on ultra‐low attachment round bottom 96‐well plates and cultured with 100 μL·well^−1^ of the 3D culture medium. After incubation for 3 days, 50 μL of the medium/well was replaced adding Matrigel Growth Factor Reduced Basement Membrane Matrix (356230, Corning, NY, USA) on top of the cells. After additional culture for 3 days, the invasion area was measured by using the image j software (National Institutes of Health, Bethesda, MD, USA). The data are presented as average values with standard deviations of at least three biological experiments; in each experiment 10 images were quantified.

### 2.14 MTS assay

Cell proliferation was analyzed by CellTiter 96® AQueous One Solution Cell Proliferation Assay (Promega, Madison, Wisconsin, USA, Cat# G3581), following the protocol by the manufacturer. The data are presented as average values with standard deviations of at least three biological experiments; each experiment consisted of 3 technical repeats.

### 2.15 ELISA

TGFβ1 was measured in the conditioned medium of *E‐cadherin*‐RFP/Py2T cells using human TGFβ Quantikine ELISA kit and mouse TGFβ1 Duoset ELISA (R&D Systems Inc., Minneapolis, MN, USA, Cat# DB100B and Cat# DY1679‐05, respectively), according to the manufacturer’s instructions. Conditioned medium was collected from approximately 80 3D mammospheres and after 1 : 10 dilution was analyzed by ELISA. For human TGFβ1 ELISA, stem cell or 2D cell culture media were used as negative controls. For mouse TGFβ1 ELISA, 2 ng·mL^−1^ human TGFβ1 was used as negative control.

### 2.16 Flow cytometry analysis

Cells cultured in the 2D condition were trypsinized and 3D cell cultures were dissociated to single cells by accutase (Gibco/Invitrogen, Thermo Fisher Scientific, Stockholm, Sweden) at 37 °C for 30 min. The cells were washed by PBS twice and re‐suspended by Brilliant Stain Buffer (Becton, Dickinson and Company, Franklin Lakes, New Jersey, USA). For cell surface marker analysis, fluorescence‐conjugated antibodies were mixed with the cells at 4 °C for 60 min. Cell surface markers were detected by fluorescence‐conjugated antibodies, which are listed in Table S6. For cell cycle analysis, the cells were mixed with RNase A (Invitrogen, Thermo Fisher Scientific, Stockholm, Sweden) at 37 °C for 30 min, and analyzed by Vybrant® DyeCycle™ Violet Stain (Invitrogen, Thermo Fisher Scientific, Stockholm, Sweden, Cat# V35003), according to the protocol by the manufacturer.

### 2.17 Time‐lapse imaging

Cells were grown and treated with 5 ng·mL^−1^ TGFβ1 on a glass‐bottom dish, and EMT time‐lapse imaging (Movie S1) was performed with an TCS SP8 microscope (Leica Microsystems, Wetzlar, Germany). The time‐lapse images were acquired every 15 min for a total imaging time of 48 h. After induction of EMT by TGFβ1 for 7 days, the cells were grown without TGFβ1 and MET imaging was performed with an ECLIPSE Ti2 microscope (Nikon, Minato, Japan). Images were acquired every 30 min with a total imaging time of 62 h (Movie S2). Five days after mammosphere formation in ultra‐low attachment condition (3D), mammospheres were grown with (complete EMT) or without (partial EMT) 5 ng·mL^−1^ TGFβ1 on a glass‐bottom dish, and time‐lapse imaging was performed with an ECLIPSE Ti2 microscope (Nikon, Minato, Japan). Images were acquired every 8 min for a total imaging time of 55 h (Movie S3).

For RFP intensity quantification of migratory cells from mammospheres, 3 cells were selected at random from each quadrant of one sphere in the differential interference contrast (DIC) channel (total 60 cells per condition). Then, the mean RFP intensity was measured in the RFP channel by image j software. All experiments were recorded using identical image acquisition conditions.

### 2.18 Extreme limiting dilution analysis (ELDA)

*E‐cadherin*‐RFP/Py2T cells were seeded on ultra‐low attachment 96‐well plates in decreasing serial dilutions (200‐1 cells/well) and cultured for 7 days. Stem cell frequency was calculated by using the ELDA program (http://bioinf.wehi.edu.au/software/elda).

### 2.19 Quantification and statistical analysis

In several experimental assays, data are presented as average determinations from biological replicates (*n* = 3 independent experiments) with standard deviation (SD) depicted as error bars. Each biological repeat was analyzed using triplicate repeats (technical repeats). The specific assays that belong to the above group include the proliferation, qRT‐PCR and luciferase assays. In contrast, the immunoblotting and immunofluorescence experiments are demonstrated using images from single, representative experiments. These experiments were repeated three or more times (*n* ≥ 3 biological repeats) in the case of immunoblotting and (*n* = 2–4 biological repeats with single technical repeats) in the case of immunofluorescence experiments. In each technical repeat of the microscopic experiments, 4‐5 independent photomicrographs were analyzed; subjective assessment was used to ascertain reproducibility among the selected photomicrographs. Statistical differences between various measurements were analyzed in the excel software based on a two‐tailed paired Student’s t test. A *P*‐value ≤0.05 was always considered as necessary for the assignment of statistical significance. Every figure legend indicates the specific *P*‐values for each specific statistical comparison. For the RNASeq transcriptome gene expression analysis, details are given in the specific method.

### 2.20 Ethical approval for animal experiments

All mouse experiments were performed with the approval of the Animal Ethics Committee of the University of Tsukuba (Ibaraki, Japan). Zebrafish experiments were conducted according to international guidelines and was approved by the local Institutional Committee for Animal Welfare (Dier Ethische Commissie (DEC)) of Leiden University Medical Center, The Netherlands.

## 3 Results

### 3.1 Analysis of different EMT cell models

**Fig. 1:**
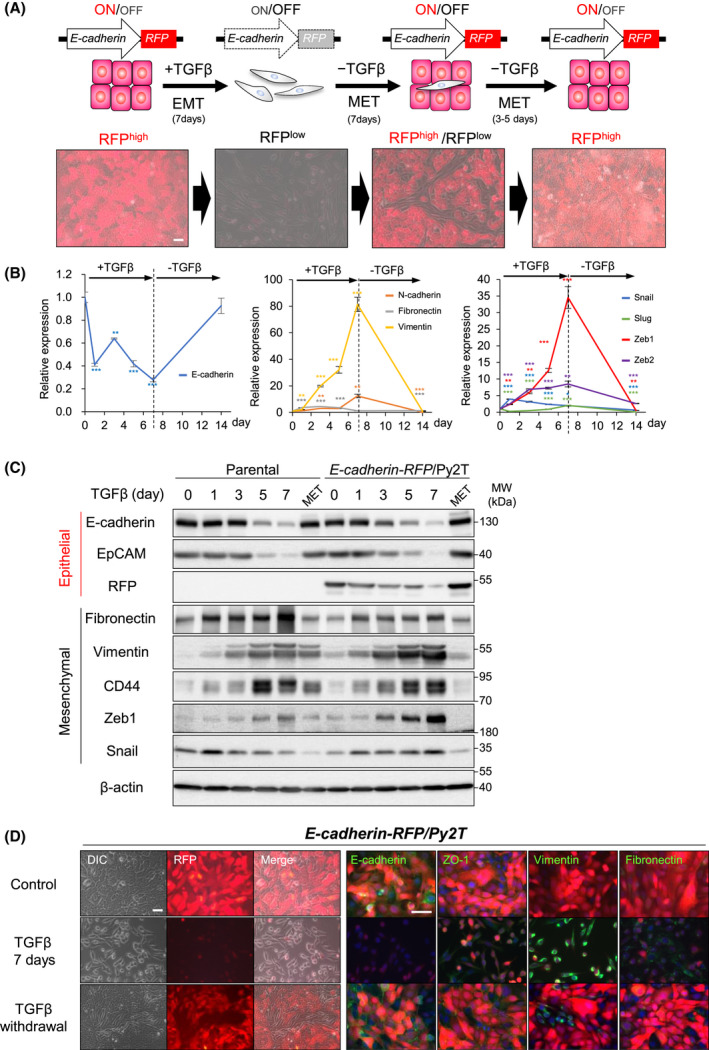
Establishment of an EMT imaging system. (A) Schematic overview of EMTimage, expressing RFP under the control of the *E‐cadherin* promoter. Differential interference contrast (DIC)/RFP fluorescence overlay images of living *E‐cadherin*‐RFP/Py2T cells during EMT/MET. EMT was induced by 5 ng·mL^−1^ TGFβ1 stimulation for 7 days and MET was induced by withdrawal of TGFβ1 for 7 days. Scale bar, 50 μm. (B) Relative expression of epithelial and mesenchymal genes normalized to *Gapdh* in *E‐cadherin*‐RFP/Py2T cells responding to TGFβ for the indicated time periods. Average values and SD from *n* = 3 biological replicates, each with technical triplicates, and *P*‐values (**P < 0.01, ***P < 0.001) after two‐tailed paired Student’s *t*‐test. (C) Immunoblot of parental Py2T and *E‐cadherin*‐RFP/Py2T cells for epithelial and mesenchymal proteins, and β‐actin as a loading control, along with molecular size markers (representative of *n* = 3 independent experiments). For original images, see Fig. S11. (D) Representative imaging of DIC, RFP, immunofluorescence staining (green) of epithelial and mesenchymal proteins and nuclear DAPI (blue) during EMT (TGFβ) and MET (TGFβ withdrawal) in *E‐cadherin*‐RFP/Py2T cells (*n* = 3 independent experiments). Scale bars, 50 μm.

To visualize EMT downstream of TGFβ, we established different imaging systems, consisting of an epithelial gene promoter *Cdh1* (*E‐cadherin* [29])*, Esrp2* (epithelial splicing regulatory protein‐2 [30]) or *miR200s* [13]) driving expression of tdTomato‐red fluorescent protein (RFP) in various mouse and human cell models. Carcinoma cell lines were screened for expression of the three reporters (Tables S1, S2). Human lung adenocarcinoma A549 and mouse breast carcinoma EpRas and Py2T cells were selected and shown to undergo TGFβ‐induced EMT (Fig. S1A–I), as previously established; human breast carcinoma MCF10A‐MII cells were excluded from the candidates because E‐cadherin mRNA/protein expression changed only weakly (Fig. S1J‐L). All cell models exhibited early induction of Snail followed by a higher magnitude and more sustained induction of ZEB1 (Fig. S1), in agreement with previous mathematical modeling studies based on human MCF10A or mouse NMuMG cells [9, 11, 12, 13, 14]. The three epithelial reporters were repressed after co‐expression of five established EMT‐TFs (Snail, Slug, ZEB1, ZEB2, Twist1) in A549, EpRas and Py2T cells (Fig. S2A‐I). Once stably transfected cell lines were established (Fig. S2J), A549 and EpRas cell clones resulted in expression of very weak or no RFP levels driven by the three promoters. In contrast, Py2T cells stably expressing *E‐cadherin*‐RFP or *Esrp2*‐RFP expressed strong fluorescence, whereas *miR200s*‐RFP cells expressed weak fluorescence, rendering the latter unsuitable (Fig. S2K). The induction of EMT by TGFβ resulted in essentially complete loss of RFP in the *E‐cadherin*‐RFP/Py2T model, whereas sparse RFP‐positive cells were evident in the *Esrp2*‐RFP/Py2T model (Fig. 1D, Fig. S2L). Furthermore, upon the withdrawal of TGFβ and culture for several days, *E‐cadherin*‐RFP/Py2T cells exhibited MET with strong RFP levels in the majority of cells, whereas *Esrp2*‐RFP/Py2T cells failed to exhibit robust MET with strong RFP re‐expression (Fig. 1D, Fig. S2L). These results made us abandon the *Esrp2*‐RFP/Py2T model and choose the *E‐cadherin*‐RFP/Py2T cells as suitable for EMT imaging.

### 3.2 Establishment and biological analysis of EMTimage

Based on the above results, we continued with the establishment of the imaging system that consisted of the mouse *Cdh1/E‐cadherin* promoter [29], driving the expression of tdTomato‐RFP in the mouse breast carcinoma Py2T cells (Fig. 1A). The stable reporter system of *E‐cadherin*‐RFP/Py2T cells was named EMTimage (Fig. 1A; Movie S1). In the following presentation of the results, we frequently use the following terms: RFP^high^ cells represent the *E‐cadherin*‐RFP/Py2T cells without treatment in 2D culture (Fig. 1A, bottom left). Spindle‐shaped cells induced after 7 days of TGFβ treatment are described as RFP^low^ cells; however, after 7 days of TGFβ treatment, the cell population may contain additional cells with a lower extent of EMT (Fig. 1A, bottom, second from the left). In addition to fluorescence, the epithelial markers E‐cadherin, EpCAM, ZO‐1 and RFP were decreased during EMT (Fig. 1B‐D). Conversely, the mesenchymal markers fibronectin and vimentin, the cancer stem cell marker CD44, the EMT‐TFs Zeb1 and Snail were all strongly increased (Fig. 1B,C). After MET was induced by the withdrawal of TGFβ, EMTimage cells re‐expressed epithelial and decreased mesenchymal gene and protein expression (Fig. 1B‐D; Movie S2). Note that 7 days of withdrawal from TGFβ generated a mixed cell population with mostly RFP^high^ and fewer RFP^low^ cells (Fig. 1A, bottom, third from the left), whereas complete MET required further prolonged culturing after withdrawal from TGFβ for an additional 3–5 days (10 to 12 days of withdrawal in total; Fig. 1A, bottom, right). It should also be noted that the *E‐cadherin*‐RFP reporter initiated its downregulation in response to TGFβ stimulation faster than the endogenous E‐cadherin protein (Fig. 1C 1 h); however, in all experiments, the RFP protein followed similar kinetics of downregulation and always required 7 days of TGFβ stimulation to exhibit maximal downregulation (Fig. 1C). It is worth noting that the mechanism of E‐cadherin and RFP protein degradation may not follow exactly the same molecular pathway, as E‐cadherin is a transmembrane protein and RFP is expressed throughout the cells. Despite this potential difference, all experiments provided data whereby RFP expression faithfully mimicked endogenous E‐cadherin expression.

**Fig. 2:**
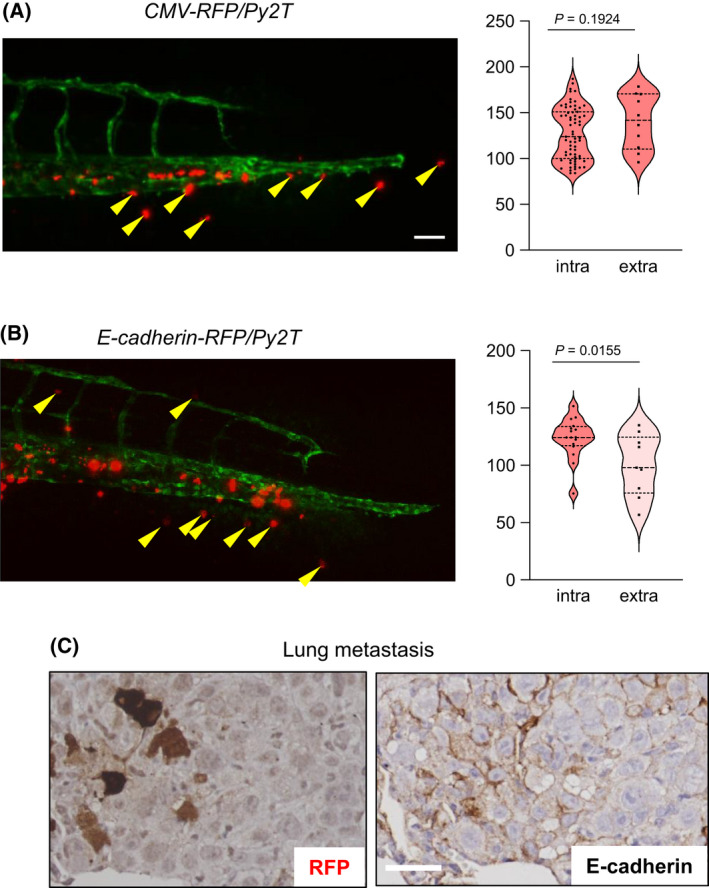
EMTimage cell invasion in the zebrafish tail and mouse lung metastasis. (A, B) Zebrafish invasion assay. Representative confocal images of zebrafish 5 days post‐implantation, with EGFP‐positive vasculature and RFP‐positive *CMV*‐RFP/Py2T (positive control) cells or *E‐cadherin*‐RFP/Py2T circulating and extravasated cells (indicated with yellow arrows). Scale bar, 50 µm. Violin plots quantifying RFP intensity of circulating (intra) tumour cells and extravasated (extra) cells and associated significance. Dotted lines indicate the median, upper and lower quartile limits. Data values from 76 (A) or 25 (B) data points from *n* = 5 biological replicates and associated significance (*P*‐value) assessed by two‐tailed paired Student’s *t*‐test. (C) Representative images of immunohistochemical staining of RFP and E‐cadherin in metastatic breast tumour in the mouse lung generated after injection of *E‐cadherin*‐RFP/Py2T cells into mouse mammary fat pads followed by spontaneous metastasis (*n* = 7 biological replicates). Scale bar, 20 μm.

EMT generates invasive cells and is usually linked to the process of cancer metastasis [1, 2]. To analyze the behaviour of EMTimage cells *in vivo*, we first used the zebrafish invasion model to analyze tumour cell invasion into the collagenous tail fin, after extravasation from the vasculature [27]. This *in vivo* assay relies on the injection of a homogeneously dispersed cell population in the fish larvae, thus precluding us from performing comparative analysis of 2D cell cultures with 3D mammospheres. As control, we used Py2T cells expressing RFP driven by a constitutive cytomegalovirus (CMV) promoter‐enhancer transcriptional unit (Fig. 2A). Upon injection of EMTimage cells in the duct of Cuvier, circulating cells maintained high RFP levels, indicating an epithelial phenotype (Fig. 2B). In contrast, cells that invaded the collagenous tail fin after extravasation lost their RFP signal, when RFP was driven by the *E‐cadherin* transcriptional unit (Fig. 2B), but not when RFP was driven by the *CMV* transcriptional control (Fig. 2A). The latter indicates that invasive EMTimage cells undergo EMT *in vivo* and further agrees with previous observations of EMT during cancer cell extravasation [40].

After transplantation of EMTimage cells in the mammary fat pads of mice and development of primary breast tumours, we also analyzed metastasis to the lungs of these animals (for detailed results of the mouse *in vivo* experiments see Section 3.7). Metastatic nodules generated in the lungs of mice carrying primary breast tumours generated by the EMTimage cells were clearly observed (Fig. 2C). Notably, such metastases contained both RFP‐positive and RFP‐negative cells (Fig. 2C). The RFP expression in the lung metastases to a large extent coincided with the corresponding positivity of cells for E‐cadherin expression. Thus, EMTimage, in addition to invasive behaviour, can sustain metastatic behaviour in transplanted mice and confirms the presence of epithelial, RFP^high^ and E‐cadherin‐positive cells in the lung metastases.

In agreement with previous evidence [41], cells exhibiting EMT had a slower cell cycle progression with more cells arrested in the G1/G0 phase (Fig. S3A‐C). After inducing the EMT/MET cycle, mixed RFP^high/low^ populations were sorted into RFP^high^ and RFP^low^ cells. RFP^high^ cells formed epithelial islands with epithelial gene expression profiles and RFP^low^ cells lost cell‐cell contacts, showed spindle‐like morphology and mesenchymal gene expression profiles (Fig. S3A,D).

### 3.3 Dynamic transcriptomic adaptation of EMTimage: epithelial, mesenchymal and reverted epithelial gene profiles

We investigated differences of total RNA expression in the three cell phenotypes, epithelial (control), mesenchymal (EMT) and reverted epithelial (MET) *via* RNAseq (Fig. S4A,B). Using EdgeR and multidimensional scaling plot analysis, we found that epithelial (control and MET) RNA profiles were clustered, whereas the profile of EMT cells was different (Fig. S4A, represented as a heat map). Scatter plot representation of the data clearly indicated a strong concordance between epithelial (control) and reverted epithelial (MET) gene profiles, which were differentiated from the mesenchymal (EMT) profiles (Fig. S4B). Gene set enrichment analysis (GSEA) of the three phenotypes showed clear positive enrichment of EMT (NES = 2.42), TGFβ signaling (NES = 1.74) and TNFα signaling (NES = 1.52) terms and negative enrichment of the p53 pathway (NES = −1.76) among the genes that were upregulated in the mesenchymal phenotype (Fig. S4C). Conversely, when scoring for genes enriched in the reverted epithelial (MET) phenotype, EMT (NES = −2.11), TGF‐β signaling (NES = −1.75) and TNFα signaling (NES = −1.63) were negatively enriched, whereas the p53 pathway (NES = 1.60) was positively enriched (Fig. S4D). Gene ontology analysis using the KEGG database showed the enrichment of cytoskeletal, Ras and Rap1 signaling, matrix‐adhesion and proteoglycan gene function terms, which were upregulated, and p53 pathway and cell‐cell contact gene function terms, which were downregulated in the mesenchymal (EMT) samples (Fig. S4E–G). Collectively, these data demonstrated that EMTimage expresses a plastic transcriptomic response to TGFβ signaling and offer a resource of differentially expressed genes that associate with each of the three phenotypes acquired by the cell model (Tables [Link], [Link], [Link], [Link], [Link]).

### 3.4 TGFβ promotes 3D mammospheres expressing RFP^low^

**Fig. 3:**
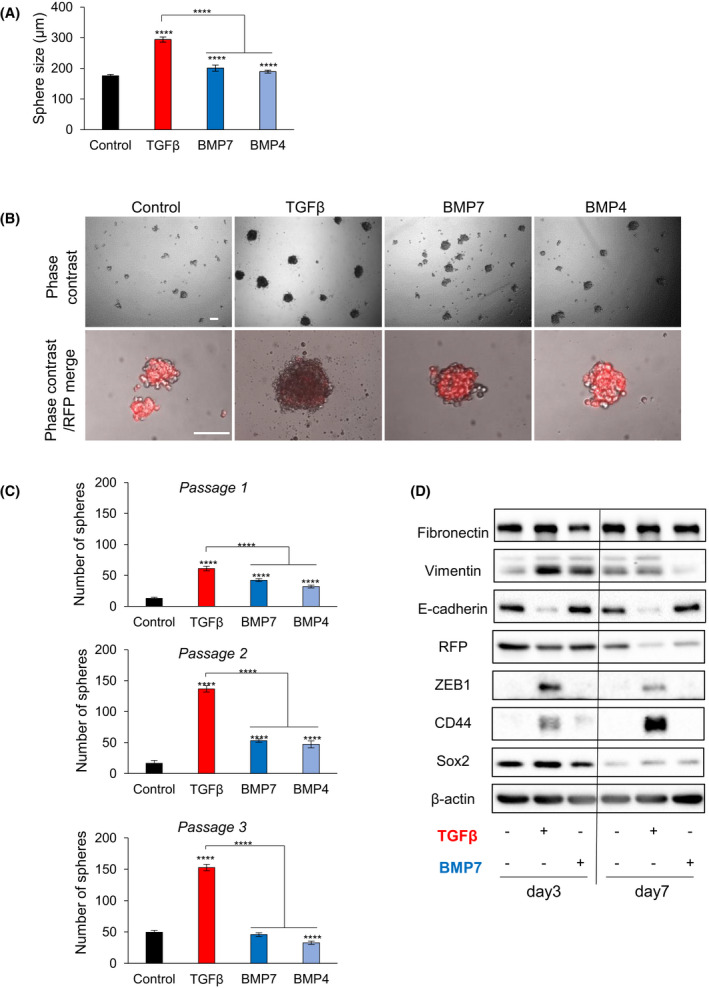
TGFβ promotes 3D mammosphere growth. (A) Mammosphere size after treatment or not of 4000 EMTimage cells with TGFβ1, BMP7, or BMP4 for 4 days in a 96‐well round bottom low‐attachment plate to obtain single spheres per well. Graph bars are colour‐coded (red, TGFβ1 (5 ng·mL^−1^); dark blue, BMP7 (100 ng·mL^−1^); light blue, BMP4 (100 ng·mL^−1^)) and show average values and SD from *n* = 13 biological replicates, each with technical triplicates, and *P*‐values (****P < 0.0005) after two‐tailed paired Student’s *t*‐test. (B) Representative DIC and RFP fluorescence overlay images of *E‐cadherin*‐RFP/Py2T cells from passage 1, in three consecutive 3D cultures (*n* = 4 independent experiments). Scale bar, 100 μm. (C) Numbers of mammospheres in the three consecutive passages of the mammosphere cells. The cells were treated under the same conditions as in panel A. Graph bars are colour‐coded (red or blue) and show average values and SD from *n* = 3 biological replicates, each with technical triplicates, and *P*‐values (****P < 0.0005) after two‐tailed paired Student’s *t*‐test. (D) Immunoblot of *E‐cadherin*‐RFP/Py2T cells cultured under 3D conditions and collected after stimulation with TGFβ1 (5 ng·mL^−1^) or BMP7 (100 ng·mL^−1^) for 3 and 5 days, followed by analyses for RFP, and epithelial, mesenchymal and stem cell proteins, as well as β‐actin as a loading control, along with molecular size markers (representative of *n* = 3 independent experiments). For original images, Fig. S11.

Since EMT links to the generation of cancer stem cells [2], we analyzed cancer cell stemness in mammosphere culture. In the first phase of these experiments, we adopted the conventional protocol of 3D culture of the cells under non‐adherent conditions using low‐attachment culture plates. As previously demonstrated for parental Py2T cells [23], EMTimage cells generated mammospheres with considerable diameter (150–200 µm, Fig. 3A). Stimulation of EMTimage with TGFβ for 4 days under the same 3D culture conditions resulted in a measurable increase of mammosphere size (Fig. 3A). It has been reported that bone morphogenetic proteins (BMPs), members of the TGFβ family, can induce MET [42, 43]. For this reason, we stimulated EMTimage with BMP4 or BMP7 during the 4‐day period (Fig. 3A), in order to evaluate two different members of the BMP family that have distinct biological effects in development and in cancer [44]. Both BMP4 and BMP7 resulted in a significant increase of mammosphere size relative to control; however, the effects of BMP4 and BMP7 stimulation were significantly smaller than that of TGFβ‐stimulation (Fig. 3A). This result possibly implies that the impact of TGFβ and BMP signaling on mammosphere growth can be differentiated from their impact on EMT/MET, as studied in 2D culture.

In order to consolidate this effect, we performed serial passages of the mammospheres in the absence or presence of TGFβ1, BMP4 or BMP7 (Fig. 3B, C). EMTimage cells generated mammospheres with the large majority of cells—if not all cells—being primarily RFP‐positive (Fig. 3B). TGFβ1 stimulation led to a distinctly higher number of mammospheres, which were built by cells expressing low or undetectable RFP levels (Fig. 3B,C). In contrast, the BMPs enhanced the number of spheres with strong RFP content (Fig. 3B,C, passage 1). TGFβ1 showed higher efficiency of mammosphere formation relative to the BMPs in each of the three consecutive passages of mammosphere cells (Fig. 3C). TGFβ1 maintained the same high efficiency even at passage 3, whereas BMPs lost their potential after passage 1 and generated the same number of mammospheres as the control nontreated condition at passage 3 (Fig. 3C). Of note, BMP4 and BMP7 did not alter the RFP content of the mammosphere cells, and these 3D cultures appeared much closer to controls (Fig. 3B), which agrees with the previously established pro‐MET action of the BMPs on breast cancer cells [42, 43]. Thus, TGFβ prominently, and BMPs less prominently, promoted stem cell frequency in EMTimage cells. Stemness measured using this 3D culture assay correlated with an RFP^low^ cell content.

TGFβ1 and BMP7 exhibited some common and some drastically different responses based on the analysis of expression of mesenchymal (fibronectin and vimentin), epithelial (E‐cadherin and RFP), EMT transcription factor (ZEB1) and breast cancer stem cell (CD44 and Sox2) proteins under the same 3D culture conditions after stimulation for 3 and 7 days (Fig. 3D). While both TGFβ and BMP induced vimentin expression at 3 days, this response was not sustained; E‐cadherin was downregulated specifically by TGFβ, but not by BMP, at both time points and this correlated best with the specific response of ZEB1 to TGFβ stimulation (Fig. 3D). In terms of stemness protein markers, CD44 exhibited a specific and highly sustainable response to TGFβ, whereas its weak response to BMP at day 3 was not sustainable (Fig. 3D). Thus, combining the 3D culture growth data with the molecular marker analysis suggests that the upregulation of ZEB1 and CD44 and downregulation of E‐cadherin best correlate with stemness and 3D mammosphere growth.

**Fig. 4:**
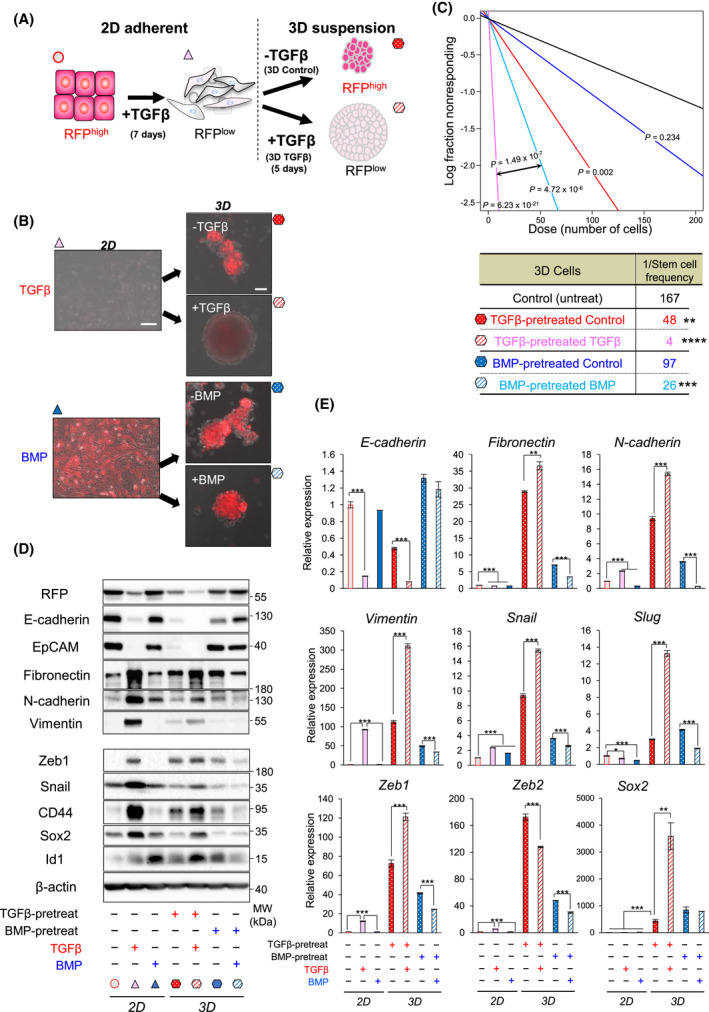
TGFβ increases EMTimage stem cell frequency. (A) Schematic drawing of the experiment. Upon TGFβ‐induced EMT of *E‐cadherin*‐RFP/Py2T cells (marked with a red circle) cultured under 2D conditions, RFP^low^ mesenchymal cells (marked by light pink triangle) were cultured in 3D. In the absence of TGFβ in 3D, the mammosphere cells underwent partial MET and became enriched in RFP^high^ cells (marked by red polka‐dotted hexagon). In the presence of TGFβ in 3D, the mammospheres remained in an RFP^low^ state and grew larger (marked by light pink striped hexagon). Note that the same colour‐coded symbols are used in panels B‐E. (B‐E) 2D cells were pre‐treated with 5 ng·mL^−1^ TGFβ1 (red symbols) or 100 ng·mL^−1^ BMP7 (blue symbols) for 7 days and then analyzed (2D), or trypsinized and seeded in 3D cultures in the absence or presence of 5 ng·mL^−1^ TGFβ1 or 100 ng·mL^−1^ BMP7 for 5 days (3D). Representative DIC and RFP fluorescence overlay images of *E‐cadherin*‐RFP/Py2T cells in 2D and 3D culture (*n* = 3 biological replicates). Scale bar, 100 μm. (C) ELDA measuring sphere‐forming frequency upon TGFβ1 or BMP7 stimulation for 7 days. The number of wells devoid of spheres (fraction nonresponding) is plotted against the number of plated cells per well (from 200 to 1 cell). Average stem cell frequency values are fitted into straight lines. Steeper slopes indicate higher frequencies of sphere‐forming cells. A table indicates average stem cell frequency per condition and associated *P*‐values (*n* = 3 biological replicates, technical octaplicates; **P < 0.01, ***P < 0.001, ****P < 10^‐6^) after χ^2^‐test followed by *P*‐value test performed to assess goodness of fit. (D) Immunoblot of *E‐cadherin*‐RFP/Py2T cells cultured and stimulated as explained in panel B, as indicated, and analyzed for RFP, and epithelial, mesenchymal and stem cell proteins, as well as β‐actin as a loading control, along with molecular size markers (representative of *n* = 3 independent experiments). For original images, Fig. S11. (E) Relative expression of the indicated genes normalized to *Gapdh* in *E‐cadherin*‐RFP/Py2T cells cultures as explained in panel B, shown as average values and SD from *n* = 3 biological replicates, each with technical triplicates, and *P*‐values (*P < 0.05, **P < 0.01, ***P < 0.001) after two‐tailed paired Student’s *t*‐test. Graph bars are colour‐coded as explained in panels A and B.

In order to scrutinize this evidence deeper and based on the result that the mesenchymal breast cancer stemness markers ZEB1 and CD44 best correlated with 3D mammosphere growth, we adopted an alternative protocol (Fig. 4A). We first generated mesenchymal RFP^low^ EMTimage cells, which were collected after a 7‐day TGFβ stimulation (called “pre‐treatment with growth factor”), and were then cultured in 3D (Fig. 4A). Once in the low‐adhesion, 3D culture, the mesenchymal RFP^low^ cells were supplemented with vehicle (control) and formed RFP^high^ cell mammospheres with fewer RFP^low^ cells, indicating that 3D culture conditions in the absence of TGFβ favour the epithelial phenotype and MET (Fig. 4B). In contrast, TGFβ‐pretreated cells that continuously received TGFβ in the 3D culture, generated large, spherical mammospheres with lower but distinct RFP expression (Fig. 4B), suggesting that even in the continuous presence of TGFβ, 3D culture conditions enrich for a cell population that exhibits partial EMT or a mixture of epithelial and mesenchymal cells. Pre‐treatment of EMTimage with BMP7 preserved the epithelial RFP^high^ phenotype; upon re‐addition of BMP7 during the 3D culture, better organized mammospheres, with higher RFP levels compared to the control condition, were observed (Fig. 4B). TGFβ stimulation strongly induced the stem cell frequency in 3D culture, whereas BMP had significant but weaker effects (Fig. 4C).

Examination of genes of the EMT and stemness programmes at the protein and mRNA level clearly established that TGFβ stimulation led to robust loss of RFP in 3D cultures, consistent with the almost complete loss of E‐cadherin and EpCAM (Fig. 4D,E). In contrast, BMP treatment preserved *E‐cadherin* mRNA and E‐cadherin and EpCAM protein expression (Fig. 4D,E), which is compatible with the observed higher RFP levels in BMP‐treated cells in 3D culture (Fig. 3B,4B). Conversely, the mesenchymal markers fibronectin, N‐cadherin and vimentin, the EMT‐TFs Snail, Slug, Zeb1 and Zeb2, and the cancer stem cell markers CD44, Sox2 and Id1 were strongly induced by TGFβ and repressed by BMP in 3D cultures (Fig. 4D,E). It is worth noting that in 2D culture many of these genes responded weakly to BMP signaling, whereas Id1 was potently induced by BMP (Fig. 4D,E), highlighting the adaptation of signaling responses to TGFβ/BMP under 3D culture conditions.

### 3.5 Cell surface EMT‐score analysis in 3D mammospheres

Cell surface proteins that characterize different stages of EMT have been reported in the studies of mouse models [19, 20, 21, 22], including the early EMT phenotype (EpCAM^low^/CD51^‐^/CD61^‐^/CD106^‐^ (triple‐negative (TN) for the last 3 markers), the partial EMT or epithelio‐mesenchymal/quasi‐mesenchymal phenotype (measured as EpCAM^low^/CD106^+^, EpCAM^low^/CD51^+^, EpCAM^low^/CD51^+^/CD106^+^, EpCAM^low^/CD51^+^/CD61^+^ or CD24^low^/CD44^high^/CD104^high^) and the complete EMT or mesenchymal phenotype (EpCAM^low^/CD51^+^/CD61^+^/CD106^+^ (triple‐positive (TP) for the last 3 markers). We examined the effect of TGFβ on the above populations (EMT‐score) by flow cytometry.

**Fig. 5:**
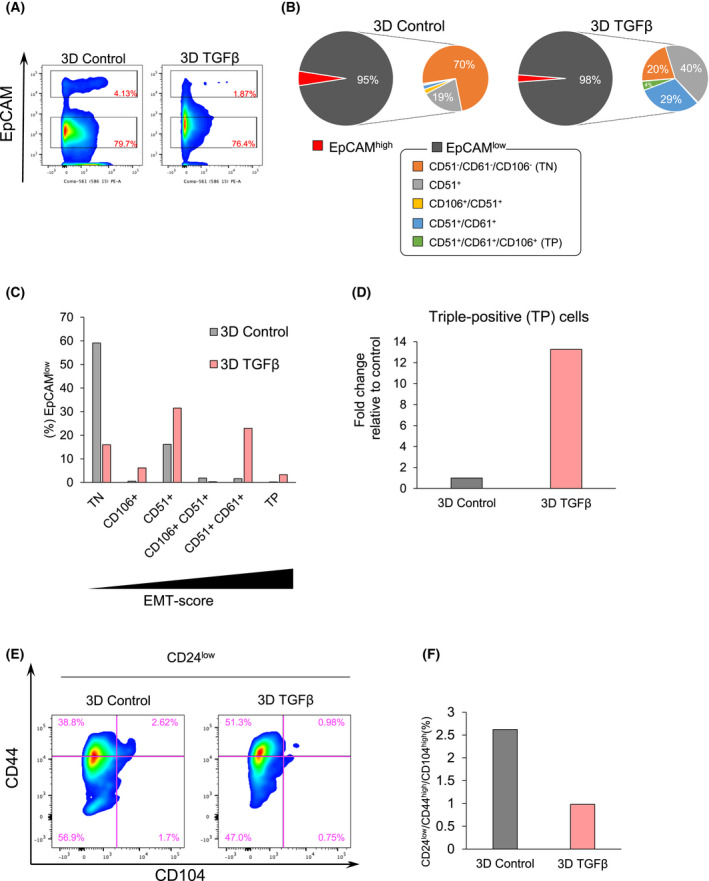
Partial EMT, EpCAM^low^/CD51^+^ and EpCAM^low^/CD51^+^/CD61^+^ cells, are preferentially induced by TGFβ under 3D culture conditions. (A) EMT‐score analysis by flow cytometry of cell surface proteins in the indicated two biological conditions and with detailed analysis in Fig. S5. EpCAM^high^ or EpCAM^low^ populations were gated. Three biological replicates were used for the analysis. (B) Pie charts illustrating the indicated colour‐coded cell populations as percentage of the total under the two biological conditions used. The cell type analysis was performed by flow cytometry of the indicated surface proteins in the EpCAM^low^ population. Detailed analysis is shown in Fig. S5. (C) Impact of TGFβ on EMT‐scores under 3D culture conditions. The data are identical to those of panel B (*n* = 3 biological replicates). Triple‐negative (TN) cells represent early EMT and correspond to EpCAM^low^/CD51^‐^/CD61^‐^/CD106^‐^; triple‐positive (TP) cells represent complete EMT and correspond to EpCAM^low^/CD51^+^/CD61^+^/CD106^+^. (D) The EpCAM^low^/triple‐positive cell population generated after TGFβ stimulation under 3D conditions is graphed relative to the control (no TGFβ) condition, which is normalized to 1. The data source is identical to that in panels B and C (*n* = 3 biological replicates). (E) Partial EMT‐score analysis by flow cytometry in the indicated two biological conditions. CD24^low^ cells were gated first, and CD44 and CD104 cell surface expression was analyzed using the indicated fluorescently‐conjugated antibodies. Three biological replicates were used for the analysis. (F) The percent of CD24^low^/CD44^high^/CD104^high^ cells is graphed for cells responding to TGFβ under 3D culture conditions. The data source is identical to that of panel E (*n* = 3 biological replicates).

In 3D mammospheres generated after TGFβ pre‐treatment without or with further TGFβ stimulation during 3D culture (Fig. 4A), most of the cells were EpCAM^low^ (Fig. 5A,B, Fig. S5A,B). The 70% EpCAM^low^/CD51^‐^/CD61^‐^/CD106^‐^ (TN, early EMT) population decreased in the presence of TGFβ to 20%, whereas TGFβ enhanced the EpCAM^low^/CD51^+^/CD61^+^/CD106^+^ (TP, complete EMT) subpopulation (Fig. 5B). Plotting the flow cytometry data as EMT‐scores defined by [19, 22], clearly indicated that TGFβ had a strong effect on the 3D mammosphere EpCAM^low^/CD51^+^/CD61^+^ (epithelio‐mesenchymal) population (Fig. 5C). Plotting the complete mesenchymal phenotype (highest EMT‐score) separately, confirmed the conclusion that TGFβ enhanced the EpCAM^low^/CD51^+^/CD61^+^/CD106^+^ (triple‐positive/TP) subpopulation under 3D conditions (Fig. 5D).

Using the alternative CD24^low^/CD44^high^/CD104^high^ signature to monitor the partial EMT population [20, 21], we first selected CD24^low^ cells, representing 9% (3D control) or 5% (3D TGFβ‐treated) of the total population. Under 3D conditions and within these CD24^low^ populations, TGFβ‐treated cells failed to enrich for the CD24^low^/CD44^high^/CD104^high^ population (0.98%; Fig. 5E). Plotting the data as an EMT‐score, clearly demonstrated the absence of an effect by TGFβ on the partial EMT phenotype under 3D conditions (Fig. 5F). The combined data show that when cells grow in 3D, the highest enriched subpopulation after TGFβ treatment is the EpCAM^low^/CD51^+^/CD61^+^, which possibly defines the enhanced mammosphere formation by TGFβ.

The same analysis was performed using EMTimage cultured in 2D (Fig. S6,S7), leading to similar results when partial EMT was analyzed using the EMT‐score defined by [19, 22], whereas the results differed when partial EMT was analyzed based on the [20, 21] protocol. Under 2D conditions, 90% of control cells were EpCAM^high^ and TGFβ stimulation converted 92% of the cells to EpCAM^low^ (Fig. S6A,B, Fig. S7A,B). Using the EMT‐score representation, TGFβ primarily enriched for the EpCAM^low^/CD51^+^ population (Fig. S6C). Furthermore, the fully mesenchymal (TP) subpopulation was larger in 2D cultures (Fig. S6D) relative to 3D conditions (Fig. 5D), reflecting the intensity of RFP in the mammospheres versus adherent cells (Fig. 4B). Using the alternative CD24^low^/CD44^high^/CD104^high^ signature, the CD24^low^ cells represented 10‐14% of the total population under 2D conditions (control and TGFβ‐treated). Within these CD24^low^ populations, 2D cells were primarily CD44^low^/CD104^low^ (Fig. S6E), whereas TGFβ stimulation enriched for CD44^high^/CD104^high^ cells (63.4%; Fig. S6F), the majority of which were RFP^low^ (Fig. S7C). Thus, under 2D conditions, TGFβ has a strong impact on the partial EMT or epithelio‐mesenchymal cell population (Fig. S6F), which was not apparent under 3D conditions (Fig. 5F). Despite this difference based on the protocol of references [20, 21], we conclude that TGFβ enriches EMTimage for the partial EMT, i.e. the epithelio‐mesenchymal subpopulation.

### 3.6 RFP^high^ but not RFP^low^ mammospheres generate mesenchymal, migratory cells

**Fig. 6:**
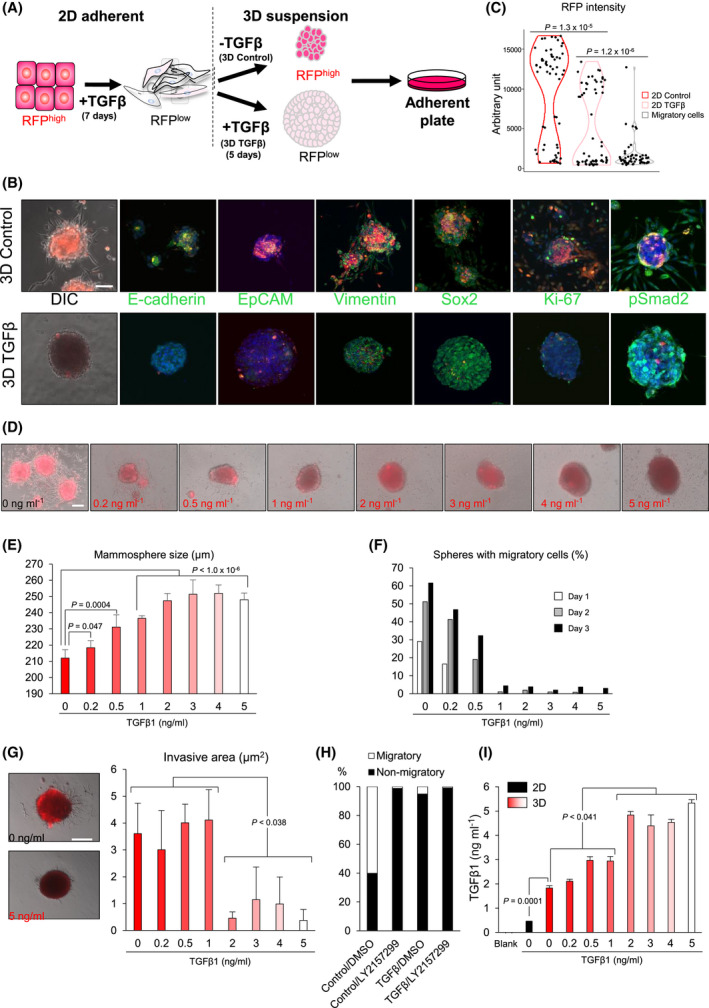
3D mammosphere growth and invasion under the influence of TGFβ. (A) Schematic drawing of the experiment as in Fig. 4A. Mammospheres were placed on adherent conditions to measure cell migration. (B) Representative DIC and fluorescence microscopy overlay images for the indicated proteins (green), RFP (red) and nuclei (DAPI, blue) of *E‐cadherin*‐RFP/Py2T mammospheres and migrating cells. Mammospheres were generated from 2D cells that were pre‐treated with 5 ng·mL^−1^ TGFβ1 for 7 days and then trypsinized and seeded in 3D cultures in the absence (3D control) or presence of 5 ng·mL^−1^ TGFβ1 (3D TGFβ) for 5 days, and finally transferred to 2D culture on glass chambers (*n* = 3 biological replicates). Scale bar, 100 μm. (C) Violin plots quantifying RFP intensity of 2D control (epithelial) and 2D TGFβ‐treated (EMT) cells as calibration controls, and in migratory cells emanating from mammospheres. Each point represents a single cell. The total cell number was *n* = 60 and significance (*P*‐value) was assessed using two‐tailed paired Student’s *t*‐test. (D) Representative DIC and fluorescence microscopy overlay images of migratory cells emanating from mammospheres under different TGFβ1 concentrations (*n* = 2 biological replicates). Scale bar, 100 μm. (E) Mammosphere size after treatment with different TGFβ1 concentrations. Data show average values and SD from *n* = 6 biological replicates per condition and significance (*P*‐value) assessed using two‐tailed paired Student’s *t*‐test. (F) Percentage of mammospheres exhibiting migratory cells emanating from spheres at the indicated time points and at different TGFβ1 concentrations. Data show average values from triplicate determinations. (G) Quantification of invasive area around the mammospheres after stimulation with the indicated TGFβ1 concentrations. DIC and fluorescence microscopy overlay images of two representative mammospheres (0 and 5 ng·mL^−1^ TGFβ1). Data show average values and SD from triplicate (*n* = 3) determinations and associated significance (*P*‐value) assessed by two‐tailed paired Student’s *t*‐test. (H) Percentage of migratory/non‐migratory cells emanating from mammospheres under the indicated conditions. The TGFβ type I receptor kinase inhibitor LY2157299 was used at 2.5 μm with DMSO as vehicle. A representative from *n* = 3 independent experiments is shown. (I) TGFβ1 concentration was measured by ELISA in the conditioned medium of the indicated conditions. Data show average values with SD from triplicate (*n* = 3) determinations and associated significance (*P*‐value) assessed by two‐tailed paired Student’s *t*‐test.

To examine whether 3D conditions that promote stem‐like features also promote cell motility, we placed mammospheres on high attachment plates and studied migratory cells emanating from the spheres (Fig. 6A). In the absence of additional exogenous TGFβ, cells inside adherent mammospheres started to re‐express RFP, E‐cadherin, EpCAM and the cell proliferation marker Ki‐67, indicating that some of the cells underwent MET and started to proliferate (Fig. 6B, 3D control). The migrating cells escaping from the mammospheres showed significantly lower RFP levels (Fig. 6B, 3D control, 6C). In contrast, mammospheres cultured with additional exogenous TGFβ did not re‐express RFP, E‐cadherin or EpCAM upon attachment to the substratum, Ki‐67 was low and sphere architecture remained intact without evidence of migration (Fig. 6B, 3D TGFβ). Migrating cells disseminated only from control spheres (RFP^high^), and not from spheres with continuous TGFβ treatment (RFP^low^) (Movie S3). When fluorescence was quantified, migratory RFP^low^ cells clustered as a single population attesting to the generation of a distinct migratory cell population with mesenchymal features (Fig. 6C). These data demonstrated that stimulation with TGFβ for long periods is important for the maintenance of stem‐like features in 3D, whereas spheres with epithelio‐mesenchymal cells generated migratory cells when exogenous TGFβ stimulation was removed. The latter allowed the transient formation of epithelial, proliferating cells that could undergo EMT again under proper context.

We examined the ability of TGFβ to promote 3D mammosphere growth with increasing doses of exogenous TGFβ; we observed a dose‐dependent increase in the size of mammospheres (Fig. 6D,E), and a dose‐dependent inhibition of mammosphere cell migration away from the spheres (Fig. 6F,G). The latter was quantified by enumerating the number of mammospheres that generated migratory cells (Fig. 6F) and by digital counting of the peri‐mammosphere surface occupied by migratory cells (Fig. 6G). This observation suggests that mammosphere size correlates with the degree of stemness (which necessitates cell proliferation) and inversely correlates with cell migration potential (which is generated once cells have undergone MET, proliferated and are thus capable of undergoing further EMT).

If the above model is correct, the MET that mammosphere cells undergo should provide a potential for these cells to re‐activate EMT using autogenous factors. We hypothesized that such an autogenous factor is TGFβ itself. A selective TGFβ type I receptor kinase inhibitor (LY2157299) completely inhibited migration from the spheres (Fig. 6H), suggesting that the migration of mesenchymal RFP^low^ cells away from the mammospheres depends on autocrine TGFβ signaling generated within the mammosphere. It is well‐known that tumour cells often secrete TGFβ. Analysis of conditioned media revealed that the mammospheres secreted 4‐fold more TGFβ1 to the culture medium compared to EMTimage cells cultured under 2D conditions (Fig. 6I). This result suggested that in 3D culture conditions, by permitting MET and cell proliferation, increased autocrine/paracrine TGFβ activity favoured stem‐like features on one hand and EMT followed by migration on the other. In order to test for this hypothesis, we analyzed the responsiveness of mammosphere cells to TGFβ during invasion. Immunofluorescence microscopy for phosphorylated Smad2 (pSmad2) allowed us to simultaneously query the extent of signaling and the types of cells that were positive for this central mediator of TGFβ signaling (Fig. 6B, Fig. S8). In control mammospheres, the pSmad2 signal was weak or negative in the nuclei of mammosphere cells, while migrating RFP^low^ cells from these mammospheres showed clear nuclear localization of pSmad2 (Fig. S8). Mammosphere treated with additional exogenous TGFβ, which could not generate migratory cells, showed larger cell numbers with higher pSmad2 expression within the mammospheres. These data confirmed that the control mammospheres that produced migratory cells were undergoing partial MET (since they expressed higher RFP signals) with less active TGFβ signaling. In conclusion, continuous presence of exogenous TGFβ stimulation of 3D mammospheres promoted stemness and inhibited migration, whereas withdrawal of the exogenous TGFβ was necessary for the generation of mesenchymal, RFP^low^ cells that could then migrate away from the mammospheres.

### 3.7 3D mammospheres responding to TGFβ differentiate between primary tumour initiation and metastatic spread *in vivo*

**Fig. 7:**
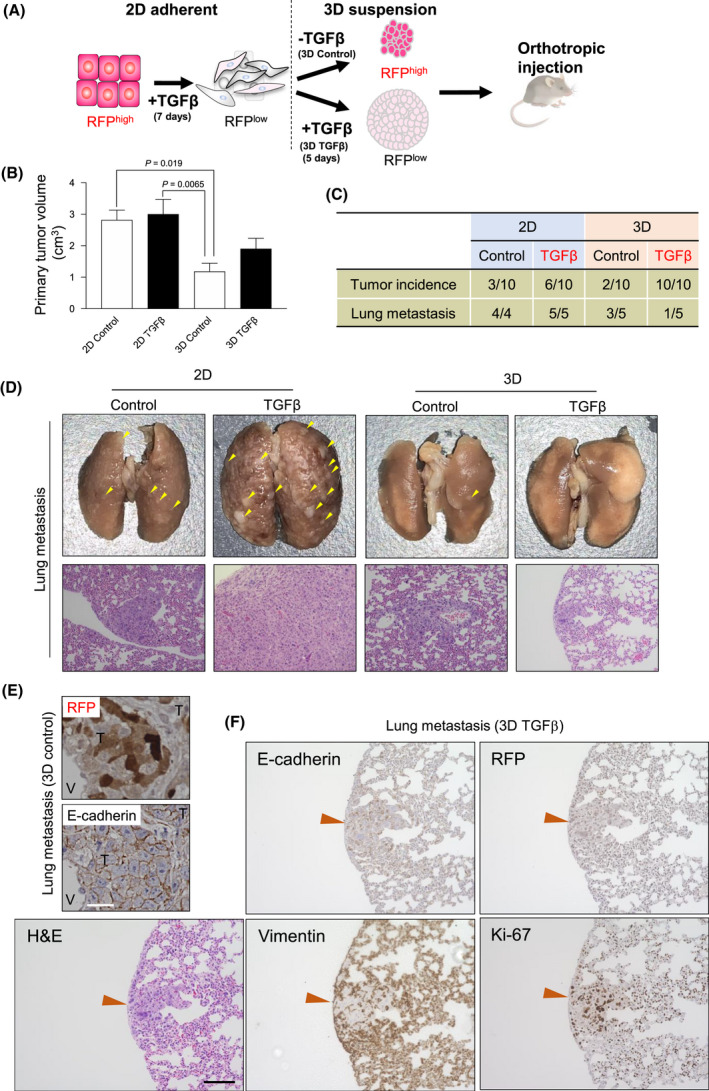
EMTimage breast tumour analysis in mice. (A) Schematic drawing of the experiment as in Fig. 4A, with mammospheres injected orthotopically in the mammary fat pads of mice. (B) Quantification of primary tumour volume 6 weeks after orthotopic injection of the indicated cells into 2 distinct sites of a mammary fat pad in each mouse. Data show average values and SD from *n* = 7 (2D control, 3D control and 3D TGFβ) or *n* = 8 (2D TGFβ) mice and associated significance (*P*‐value) assessed by two‐tailed paired Student’s *t*‐test. (C) Primary tumour and lung metastasis incidence in the indicated conditions. (D) Representative images of lung metastases (out of *n* = 13 lungs analyzed). Upper, macroscopic images of whole lungs with metastatic nodules (yellow arrowheads) generated by the indicated conditions. Scale bar, 0.5 cm. Lower, H&E staining of single metastatic nodules. Scale bar, 100 μm. (E) Representative images of immunohistochemical staining of E‐cadherin and RFP in metastatic breast tumour in the lung, generated by injection of 3D control cells into mouse mammary fat pads (*n* = 3 independent lungs analyzed). The tumour (T), nearby stromal area (demarcated by dotted lines and verified based on extracellular matrix content) and a blood vessel (V) are indicated. Scale bar, 20 μm. (F) The single lung metastasis in only one mouse (see panel C), observed 6 weeks after the injection of 3D TGFβ cells. H&E staining of the single lung metastatic nodule (orange arrowhead) along with immunohistochemistry for the indicated proteins in adjacent serial sections. Scale bar, 50 μm.

We used orthotopic transplantations in nude mice to analyze primary tumour growth and metastasis to lungs (Fig. 7A). In addition, we wanted to compare EMTimage to the parental Py2T cell model [23] in terms of the tumour features *in vivo*. Nude mice were used instead of a mouse strain syngeneic to the Py2T cells, because it was previously reported that nude mice generate primary tumours with 100% efficiency after mammary fat pad injection of the parent Py2T cells, compared to syngeneic immunocompetent FVB/N mice that generate tumours less frequently [23]. In order to perform side‐by‐side comparisons, we transplanted 2D control (RFP^high^), 2D TGFβ‐stimulated (RFP^low^), 3D control mammosphere (RFP^low^) and 3D TGFβ‐stimulated mammosphere (RFP^low^) cells into mouse mammary fat pads. Primary tumours derived from 2D cells gave rise to larger tumours compared to 3D cells regardless of TGFβ treatment (Fig. 7B). Notably, 3D TGFβ‐stimulated cells showed highest tumour initiating capacity (10/10 mice), followed by 2D TGFβ‐stimulated (6/10), 2D Control (3/10) and 3D Control (2/10) (Fig. 7C). Thus, TGFβ‐stimulated 3D mammospheres exhibited enhanced stemness features *in vitro* (Fig. 4) and highest tumour initiating potential (Fig. 7C).

Immunohistochemistry of primary tumours confirmed that 2D control cells gave rise to tumours that contained a fair proportion of RFP‐positive cells intermixed with RFP‐negative cells, whereas all other conditions showed low or very low RFP content, correlating directly with tumour cell E‐cadherin expression and inversely with mesenchymal cell protein vimentin expression (Fig. S9A,B). Primary tumour size (Fig. 7B) correlated with intratumoral Ki‐67 expression, which was consistently lower in 3D mammosphere‐derived tumours (Fig. S9A). In agreement with a previous report [23], the primary tumours formed by EMTimage cells were characterized by a tissue architecture of elongated RFP‐negative/vimentin‐positive cells (Fig. S9A,B), streaming among RFP‐positive tumour cells (Fig. S9A). Primary tumours generated from 2D and 3D TGFβ cells exhibited a larger vimentin‐positive number of cells, which were also more elongated and often arranged in parallel streaks, suggesting that EMT was evident within the primary tumour when the xenotransplanted cells were pre‐treated with TGFβ prior to injection in the mammary fat pads (Fig. S9B).

We then analyzed the ability of EMTimage cells to form metastases. Primary tumours of TGFβ‐stimulated 2D cells showed significantly higher frequency of lung metastasis (2D TGFβ), whereas tumours from TGFβ‐stimulated 3D mammospheres (3D TGFβ) showed no macro‐metastasis (Fig. 7C,D, Fig. S10). A single (1/5) mouse carrying primary tumours from 3D TGFβ mammospheres developed only one small (150 μm in diameter) micro‐metastasis in the pleura, which was clearly detectable only after sectioning and H&E staining of the whole lung (Fig. 7D, Fig. S10). In contrast, 3/5 mice implanted with control 3D mammospheres showed multiple microscopic metastatic tumours (Fig. 7D). The largest number of lung metastases per lung and in all animals tested were derived from 2D TGFβ cells (Fig. 7D).

It has been firmly established that vascular invasion detected by histopathological analysis correlates with cancer metastasis or prognosis in breast cancer patients [45, 46]. In accordance with these previous reports, vascular invasion was readily detectable in mice carrying tumours derived from TGFβ‐stimulated 2D cells, by staining for the endothelial marker CD31 in the primary tumours (Fig. S9C). On the other hand, vascular invasion was not detected in primary tumours from other conditions including TGFβ‐stimulated 3D mammospheres, correlating with the low incidence of lung metastasis. As expected, no obvious differences were observed among tumours of the four biological conditions tested in terms of the tumour vasculature architecture stained by the anti‐CD31 antibody (Fig. S9C).

Histochemical analysis of metastatic lung nodules revealed a variable degree of RFP‐positive cells, indicating that MET had taken place during colonization (Fig. 7E). Increased staining for E‐cadherin confirmed this result (Fig. 7E). When single cells were inspected in the metastatic nodules, the RFP staining varied between intensely stained, intermediate and poorly stained cells (Fig. 7E), suggesting that lung metastases exhibited a diverse degree of MET. We analyzed the EMT/MET status of the single micrometastasis generated by the 3D TGFβ cells (Fig. 7F) that failed to exhibit migration in 3D culture conditions (Fig. 6). The majority of the breast tumour cells in this lung metastasis expressed detectable E‐cadherin and RFP and lacked vimentin, although the surrounding lung cells in the host organ were vimentin‐positive (Fig 7F). The same breast epithelial tumour cells in the lung metastasis scored positive for the proliferation marker Ki‐67, suggesting that proliferation of the micronodule was linked to the epithelial phenotype, presumably generated after MET (Fig. 7F).

## 4 Discussion

In the present communication, we show that pretreatment of breast cancer cells with TGFβ promotes the formation of mammospheres with RFP^high^ cells, exhibiting partial MET; the stemness of the mammosphere cells was further enhanced by subsequent stimulation with TGFβ in 3D cultures, leading to enhanced tumour initiating capacity. Under the same 3D conditions, mammospheres with partial MET readily formed mesenchymal migratory cells with very low RFP content. Importantly, further stimulation with exogenous TGFβ prohibited migration of the latter cells from the mammospheres.

The plasticity of the EMTimage was clearly demonstrated by whole transcriptome analysis, showing that the mesenchymal phenotype gene profile was replaced by an epithelial profile once MET was achieved (Fig. S4). The transcriptomic analysis confirmed the identification of EMT and TGFβ signaling gene ontologies among the top groups of genes that were differentially expressed in mesenchymal cells, and the p53‐regulated network among the top groups of genes expressed in epithelial cells prior to EMT or after MET (Fig. S4). This rich resource of adaptations in gene expression is currently harnessed in deeper detail.

The evidence described here suggests that EMTimage preserves the features of its parental cell model Py2T [23], undergoing EMT in response to TGFβ in culture and forming primary tumours in transplanted mice (Figs. 1,7). EMTimage cells formed detectable and easy to score lung metastases (with the exception of 3D TGFβ cells), which is a deviation from the lack of lung metastases reported in the parental Py2T model [23], possibly reflecting different host mouse strains.

The cellular context within a tissue/cell population provides means to modulate the EMT/MET response (Fig. 6A, 7A). When prolonged treatment of mammary epithelial cells with TGFβ was studied in 2D cultures, evidence for a stabilized EMT that generated mesenchymal cells was reported [47, 48, 49]. Yet, under such conditions, the presence of small epithelial populations, indicative of MET, was evident [48]. Here we show that EMT‐derived mesenchymal, RFP^low^ cells, after cell divisions, form mammospheres consisting of epithelial, RFP^high^ cells (Fig. 4). It remains to be established, whether the larger mammosphere size induced by TGFβ reflects enhanced cell proliferation, reduced apoptotic rates or consecutive rounds of EMT/MET taking place within the mammosphere. Under 3D conditions, TGFβ preferably supports the formation of mammospheres and enhances stemness (Figs. 3,4), resembling the impact that stabilized EMT has on mammosphere formation by immortalized human mammary HMLE cells [49]. These observations are compatible with studies that established links between EMT induced by EMT transcription factor overexpression and tumour cell stemness [50, 51]. The stemness phenotype induced by TGFβ translated to breast cancer initiation, since best tumour incidence was scored by mammospheres stimulated with TGFβ (Fig. 7). The response of E‐cadherin‐RFP/Py2T cells in this respect is similar to the response of claudin‐low and triple‐negative breast cancer cells to TGFβ, which respond by exhibiting a pro‐stemness gene expression pattern, and in which TGFβ receptor antagonists effectively block mammosphere and tumour incidence [52, 53]. Equivalent pro‐stemness responses to TGFβ have been reported in embryonic, adult stem cells and stem cells of brain or pancreatic tumours [54, 55, 56].

Cells undergoing EMT in 2D culture showed increased motility, which was also seen when cells in 3D mammospheres adhered and migrated (Fig. 6). Mammospheres that exhibited MET also showed high potential of migration; this observation is consistent with the notion that the EMT‐invasive program is best adapted to partial EMT that generates epithelio‐mesenchymal cells in the mammosphere, which are then able to undergo more complete EMT (Fig. 6). Interestingly, cell motility away from adhering mammospheres depended on autocrine TGFβ signaling (Fig. 6H). This correlated with a higher pSmad2 content in the migratory RFP^low^ cells (Fig. 6B, Fig. S8). The mechanism that unleashes the autocrine TGFβ activity remains to be elucidated, but may be linked to the adherence of mammospheres to an extracellular surface.

The evidence for an epithelio‐mesenchymal phenotype in 3D mammospheres is compatible with fate‐mapping experiments of mouse tumours with invasive and metastatic capacities that demonstrated a requirement for partial EMT (hybrid epithelial/mesenchymal phenotype) [19, 20, 21, 22]. Using partial EMT surface marker analysis, we identified the full spectrum of phenotypes between EpCAM^low^/CD51^‐^/CD61^‐^/CD106^‐^ (early EMT) and EpCAM^low^/CD51^+^/CD61^+^/CD106^+^ (complete EMT) cells and also clear enrichment for CD24^low^/CD44^high^/CD104^high^ (partial EMT) cells (Fig. 5, Fig. S5–S7). This analysis points to strong effects of TGFβ on the intermediate EpCAM^low^/CD51^+^ population of the partial EMT. These findings are compatible with previous mathematical analysis of TGFβ‐induced EMT [11, 12, 13, 14], including the model of hysteretic EMT, which postulates that the loss of E‐cadherin during EMT requires prolonged stimulation by TGFβ and takes place abruptly upon reaching a certain threshold of signaling controlled by the negative feedback loop of the micro‐RNA miR200 [9]. According to the hysteretic EMT model, cancer stemness is generated during the period prior to E‐cadherin loss and only cells that preserve their partial EMT (E‐cadherin‐positive) phenotype contribute to lung metastasis [9]. We suggest that mammosphere cells in 3D, generated from TGFβ‐stimulated 2D mesenchymal cells, suppress the hysteretic EMT phenotype, whereas 2D cells treated with TGFβ resulted in an efficient dissemination of primary tumours to metastatic nodules in the lung (Fig. 7). Under 3D conditions, a TGFβ type I receptor kinase inhibitor suppressed cell migration from mammospheres (Fig. 6). This is compatible with *in vivo* studies of invasion of breast tumours, where only mesenchymal cells exhibited overt invasion, whereas both epithelial and mesenchymal cells were associated with tumour stemness due to the plastic interconversion of one phenotype to the other [57]. Yet, additional stimulation of mammospheres with TGFβ dose‐dependently promoted sphere growth and interfered with cell migration emanating from the mammospheres (Fig. 6). We hypothesize that 3D mammospheres exhibit a dynamic interconversion between RFP^high^ and RFP^low^ cells, as visualized also in time‐lapse videos (Movie S3), and under such conditions, TGFβ favours mammosphere growth because of the higher responsiveness of the RFP^high^ cells (Fig. S8). Thus, mammosphere re‐exposure to exogenous TGFβ enhances stemness and corresponding tumour initiating capacity, but does not support *in vitro* invasiveness or metastasis to the lung.

## 5 Conclusion

In conclusion, we propose a model for the multi‐faceted actions of TGFβ. In 2D cultures, TGFβ signaling induces EMT. Once mesenchymal cells are given the chance to proliferate and build mammospheres, partial MET takes place, generating a balanced epithelio‐mesenchymal population with stem‐like features, good tumour initiating potential and highly efficient metastasis to the lung. The mammosphere cells, when given the chance to adhere to a matrix, perform further EMT, partially dependent on autocrine TGFβ activity. The EMTimage cell model provides a useful tool that can be used to differentiate pro‐invasive from pro‐stemness phenotypes in breast cancer biology.

## Conflict of interest

The authors declare no conflicts of interest.

### Peer review

The peer review history for this article is available at https://publons.com/publon/10.1002/1878‐0261.13215.

## Authors’ contributions

YT and TI conceived the project; YT, YOh, YOk, and AM designed the experiments; YT, YOh, YOk, JR, and JE acquired the data; YT, YOh, YOk, SY, JE, MES, PtD, KM, AH, CHH, and AM analyzed the data; YT, YOh, JE, MES, PtD, KM, AH, TI, MK, CHH, and AM interpreted the data; YT drafted and AM with YOh finalized the article. All authors critically revised the article and approved its submission for publication.

## Supporting information


**Movie S1.** Time‐lapse EMT imaging of *E‐cadherin*‐RFP/Py2T cells.

**Movie. S2.** Time‐lapse MET imaging of *E‐cadherin*‐RFP/Py2T cells.

**Movie S3.** Time‐lapse imaging of *E‐cadherin*‐RFP/Py2T mammospheres during adhesion that elicits cell migration.

**Fig. S1.** EMT induction in candidate cancer cell lines.
**Fig. S2.** Screening of epithelial promoters in cancer cell lines.
**Fig. S3.** RFP^high^ cells express epithelial and RFP^low^ cells express mesenchymal genes.
**Fig. S4.** Transcriptome of the EMT and MET cell states.
**Fig. S5.** EMT‐score analysis by flow cytometry after TGFβ stimulation in 2D and 3D cultures.
**Fig. S6.** Partial EMT, EpCAM^low^/CD51^+^ or CD24^low^/CD44^high^/CD104^high^ cells are preferentially induced by TGFβ under 2D culture conditions.
**Fig. S7.** EMT‐score analysis by flow cytometry after TGFβ stimulation in 2D cultures.
**Fig. S8.** Analysis of TGFβ signaling in mammospheres.
**Fig. S9.** EMTimage primary tumor analysis.
**Fig. S10.** EMTimage lung metastasis analysis.
**Fig. S11.** Unprocessed immunoblots.
**Table S1**. Candidate cancer cell lines for EMT imaging.
**Table S2**. Cell lines used, their sources and specification.
**Table S3**. List of chemicals, peptides and recombinant proteins.
**Table S4**. List of all recombinant DNA plasmids used.
**Table S5**. List of oligonucleotides used.
**Table S6**. List of antibodies used with dilution factors and application (immunoblot, immunofluorescence, immunohistochemistry or flow cytometry (flow)

**Table S7**. Excel file with the list of differentially expressed genes in cells, related to the data of Fig. S4.

**Table S8**. Excel file (FC_Cell_FDR) with the list of differentially expressed genes in cells, related to the data of Fig. S4B.

**Table S9**. Excel file (EMT_GSEA_h.all.v7.1.entrez.gmt_EMT) with the GSEA data of differentially expressed cellular RNAs from EMT versus control cells, related to the data of Fig. S4C.

**Table S10**. Excel file (METvsEMT_GSEA_h.all.v7.1.entrez.gmt_EMT) with the GSEA data of differentially expressed cellular RNAs from MET vs EMT cells, related to the data of Fig. S4D.

**Table S11**. Excel file (KEGG_CellSimilar_Down) with the GO analysis data of differentially down‐regulated cellular RNAs from EMT cells, related to the data of Fig. S4F, G.

## Data Availability

The RNA sequencing data are deposited in Arrayexpress, EBI, UK under accession number E‐MTAB‐97509. No relevant code has been developed in this paper. Cell lines generated in this study can be made available upon request to the corresponding author.
