# Supplementary material for: TGFβ selects for pro‐stemness over pro‐invasive phenotypes during cancer cell epithelial–mesenchymal transition
Source: Mol Oncol. 2022 Apr 10;16(12):2330–54. doi: 10.1002/1878-0261.13215 (PMC9208077; doi:10.1002/1878-0261.13215)
Supplement: Supplementary file 4 — Fig. S1. EMT induction in candidate cancer cell lines. Fig. S2. Screening of epithelial promoters in cancer cell lines. Fig. S3. RFPhigh cells express epithelial and RFPlow cells express mesenchymal genes. Fig. S4. Transcriptome of the EMT and MET cell states. Fig. S5. EMT‐score analysis by flow cytometry after TGFβ stimulation in 2D and 3D cultures. Fig. S6. Partial EMT, EpCAMlow/CD51+ or CD24low/CD44high/CD104high cells are preferentially induced by TGFβ under 2D culture conditions. Fig. S7. EMT‐score analysis by flow cytometry after TGFβ stimulation in 2D cultures. Fig. S8. Analysis of TGFβ signaling in mammospheres. Fig. S9. EMTimage primary tumor analysis. Fig. S10. EMTimage lung metastasis analysis. Fig. S11. Unprocessed immunoblots. Table S1. Candidate cancer cell lines for EMT imaging. Table S2. Cell lines used, their sources and specification. Table S3. List of chemicals, peptides and recombinant proteins. Table S4. List of all recombinant DNA plasmids used. Table S5. List of oligonucleotides used. Table S6. List of antibodies used with dilution factors and application (immunoblot, immunofluorescence, immunohistochemistry or flow cytometry (flow) [file MOL2-16-2330-s007.pdf]

Supporting Information

Supporting tables

Cell lines	Species	Organ	EMT	Metastasis	Ref.
A549	Human	Lung	Yes	Yes	[1]
HMEC	Human	Breast	Yes	No	[2]
HMLE	Human	Breast	Yes	No	[3]
MCF10A MII	Human	Breast	Yes	No	[4]
MCF10A MIII	Human	Breast	No (Mesenchymal)	Yes	[4]
MDA-MB-231	Human	Breast	No (Mesenchymal)	Yes	[5]
EpRas	Mouse	Breast	Yes	Yes	[6]
Py2T	Mouse	Breast	Yes	Yes	[7]

Table S1. Candidate cancer cell lines for EMT imaging. The ability to undergo EMT in response to TGFβ after incubation for 7 days is summarized with the information on species and organ of origin for the indicated cell lines. Metastatic potential published in the corresponding reference is also enlisted.

Supporting references

1 Kubota, SI, Takahashi, K, Nishida, J, Morishita, Y, Ehata, S, Tainaka, K, Miyazono, K & Ueda, HR. (2017) Whole-body profiling of cancer metastasis with single-cell resolution. *Cell Rep* **20**, 236-250.

2 Elenbaas, B, Spirio, L, Koerner, F, Fleming, MD, Zimonjic, DB, Donaher, JL, Popescu, NC, Hahn, WC & Weinberg, RA. (2001) Human breast cancer cells generated by oncogenic transformation of primary mammary epithelial cells. *Genes Dev* **15**, 50-65.

3 Mani, SA, Guo, W, Liao, MJ, Eaton, EN, Ayyanan, A, Zhou, AY, Brooks, M, Reinhard, F, Zhang, CC, Shipitsin, M *et al.* (2008) The epithelial-mesenchymal transition generates cells with properties of stem cells. *Cell* **133**, 704-715.

4 Santner, SJ, Dawson, PJ, Tait, L, Soule, HD, Eliason, J, Mohamed, AN, Wolman, SR, Heppner, GH & Miller, FR. (2001) Malignant MCF10CA1 cell lines derived from premalignant human breast epithelial MCF10AT cells. *Breast Cancer Res Treat* **65**, 101-110.

5 Yin, JJ, Selander, K, Chirgwin, JM, Dallas, M, Grubbs, BG, Wieser, R, Massagué, J, Mundy, GR & Guise, TA. (1999) TGF-β signaling blockade inhibits PTHRP secretion by breast cancer cells and bone metastases development. *J Clin Invest* **103**, 197-206.

6 Oft, M, Heider, KH & Beug, H. (1998) TGFβ signaling is necessary for carcinoma cell invasiveness and metastasis. *Curr Biol* **8**, 1243-1252.

7 Waldmeier, L, Meyer-Schaller, N, Diepenbruck, M & Christofori, G. (2012) Py2T murine breast cancer cells, a versatile model of TGFβ-induced emt in vitro and in vivo. *PLoS One* **7**, e48651.

Cell line	Source	Specification
A549 human lung adenocarcinoma	American Type Culture Collection	ATCC Cat# CRM-CCL-185, RRID:CVCL_0023
HMEC human mammary epithelial cells	Thermo Fisher Scientific/Lonza/Clonetics	Cat# CC-2551
HMLE human mammary epithelial cells	Whitehead Institute for Biomedical Research, Cambridge, MA, USA	Dr. Robert Weinberg, gift
MCF10AT1k.cl2 (MII) human transformed breast epithelial cells	Barbara Ann Karmanos Cancer Institute, Detroit, MI, USA	Dr. Robert J. Pauley, gift
MCF10CA1h (MIII) human metastatic breast cancer cells	Barbara Ann Karmanos Cancer Institute, Detroit, MI, USA	Dr. Robert J. Pauley, gift
MDA-MB-231 human breast carcinoma cells	American Type Culture Collection	ATCC Cat# HTB-26, RRID:CVCL_0062
EpRas mouse mammary gland epithelial EpH4 cells transformed with mutant H-Ras	Institute of Molecular Pathology, Vienna, Austria	Dr. Hartmut Beug, gift
Py2T mouse breast carcinoma	University of Basel, Basel, Switzerland	Dr. Gerhard Christofori, gift

Table S2. Cell lines used, their sources and specification.

Reagent	Source	Specification
Accutase Cell Dissociation Reagent	Thermo Fisher Scientific	Cat# A11105-01
BMP4	Peprotech EC Ltd	Cat# 120-105
BMP7	Sanofi-Genzyme Research	Dr. Kuber Sampath, gift
Brilliant stain buffer	BD Biosciences-Europe	Cat# 563794
Cholera toxin	Sigma-Aldrich AB	C8052-1MG
Cisplatin	Sigma-Aldrich AB	Cat# 232120
Cyclophosphamide	Tronto Research Chemicals	Cat# C988580
Dimethyl-sulfoxide (DMSO)	Sigma-Aldrich AB	Cat# D2650
Doxorubicin	Sigma-Aldrich AB	Cat #D1515
Dulbecco's modified eagle medium (DMEM)	Sigma-Aldrich AB	Cat# D5796
DMEM/F12	Sigma-Aldrich AB	Cat# D6421
EcoRI-HF restriction enzyme	New England Biolabs Inc.	Cat# R3101L
EGF	Peprotech EC Ltd	Cat# AF-100-15
Fetal bovine serum (FBS)	Biowest, Almeco A/S	Cat# S1810
FGF-basic (154 a.a.)	Peprotech EC Ltd	Cat# 100-18B
5-FU	Sigma-Aldrich AB	Cat# F6627
Geneticin	Thermo Fisher Scientific	Cat# 11811-031
HEPES solution	Sigma-Aldrich AB	Cat# H0887
Hydrocortisone solution	Sigma-Aldrich AB	Cat# H6909-
Insulin	Thermo Fisher Scientific	Cat# 12585014
L-glutamine	Sigma-Aldrich AB	Cat# G8540
Lipofectamine 3000	Thermo Fisher Scientific	Cat# L3000-015
LY2157299	Sigma-Aldrich AB	Cat# 700874-72-2
Matrigel basement membrane matrix grouth factor reduced	Corning	Cat# 356230
MfeI restriction enzyme	New England Biolabs Inc.	Cat# R0589L
NotI-HF restriction enzyme	New England Biolabs Inc.	Cat# R3189L
Penicillin-streptomycin solution	Sigma-Aldrich AB	Cat# P0781
Protease inhibitor cocktail	Roche Diagnostics Scandinavia AB	Cat# 11873580001
PureLink RNase A	Thermo Fisher Scientific	Cat# 12091021
Taxol	Sigma-Aldrich AB	Cat# T7402
Tetramethylrhodamine-isothiocyanate-conjugated phalloidin	Sigma-Aldrich AB	Cat# P1951
TGFβ1 (human)	PeproTech EC Ltd	Cat# 100-21
TRIzol	Thermo Fisher Scientific	Cat# 15596-018
Trypsin-EDTA	Sigma-Aldrich AB	Cat# T3924
XhoI restriction enzyme	New England Biolabs Inc.	Cat# R0146L

Table S3. List of chemicals, peptides, and recombinant proteins.

Reagent	Source	Specification
$CAGA_9$-luc	Ludwig Institute for Cancer Research, Uppsala, Sweden	Dr. Peter ten Dijke (author)
E-cadherin-luc	Yamanashi University, Japan	Dr. Masao Saitoh, gift
ESRP2-luc	Yamanashi University, Japan	Dr. Masao Saitoh, gift
miR-200s-luc	University of South Australia, North Terrace, Adelaide, Australia	Dr. Gregory J. Goodall, gift
pcDNA3	Ludwig Institute for Cancer Research, Uppsala, Sweden	Dr. Carl-Henrik Heldin (author)
pcDNA3-Flag	Ludwig Institute for Cancer Research, Uppsala, Sweden	Dr. Carl-Henrik Heldin (author)
pcDNA3-Snail-HA	Yamanashi University, Japan	Dr. Masao Saitoh, gift
pcDNA3-Slug-HA	Yamanashi University, Japan	Dr. Masao Saitoh, gift
pcDNA3-Flag-ZEB1	Yamanashi University, Japan	Dr. Masao Saitoh, gift
pcDNA3-Flag-ZEB2	Yamanashi University, Japan	Dr. Masao Saitoh, gift
pcDNA3-Flag-Twist	Yamanashi University, Japan	Dr. Masao Saitoh, gift
pGL4.74	Promega	
pEF1α-tdTomato	Ehime University, Japan	Dr. Takeshi Imamura (author)

Table S4. List of all recombinant DNA plasmids used.

Reagent	Source	Specification
Oligonucleotides for RT-qPCR		
Human *E-CADHERIN*<br>Fw: TACGCCTGGGACTCCACCTA	Eurofins	Custom-made
Human *E-CADHERIN*<br>Rev: CCAGAAACGGAGGCCTGAT	Eurofins	Custom-made
Human *FIBRONECTIN*<br>Fw: CATCGAGCGGATCTGGCCC	Eurofins	Custom-made
Human *FIBRONECTIN*<br>Rev: GCAGCTGACTCCGTTGCCCA	Eurofins	Custom-made
Human *GAPDH*<br>Fw: GGAGTCAACGGATTTGGTCGTA	Eurofins	Custom-made
Human *GAPDH*<br>Rev: GGCAACAATATCCACTTTACCA	Eurofins	Custom-made
Human *N-CADHERIN*<br>Fw: CCTGCTTCAGGCGTCTGTAGA	Eurofins	Custom-made
Human *N-CADHERIN*<br>Rev: TCATGCACATCCTTCGATAAGA<br>CT	Eurofins	Custom-made
Human *SLUG*<br>Fw: AGACCCTGGTTGCTTCAAGGA	Eurofins	Custom-made
Human *SLUG*<br>Rev: CTCAGATTTGACCTGTCTGCAAA	Eurofins	Custom-made
Human *SNAIL*<br>Fw: CACTATGCCGCGCTCTTTC	Eurofins	Custom-made
Human *SNAIL*<br>Rev: GCTGGAAGGTAAACTCTGGATTA<br>GA	Eurofins	Custom-made
Human *VIMENTIN*<br>Fw: CGGGAGAAATTGCAGGAGGA	Eurofins	Custom-made
Human *VIMENTIN*<br>Rev: AAGGTCAAGACGTGCCAGAG	Eurofins	Custom-made
Human *ZEB1*<br>Fw: GAAAATGAGCAAAACCATGATC<br>CT	Eurofins	Custom-made
Human *ZEB1*<br>Rev: CCCTGCCTCTGGTCCTCTTC	Eurofins	Custom-made
Human *ZEB2*<br>Fw: CACGATCCAGACCGCAATTA	Eurofins	Custom-made
Human *ZEB2*<br>Rev: CATCGCGTTCCTCCAGTTTT	Eurofins	Custom-made
Human/Mouse *TWIST*<br>Fw: CGGGTCATGGCTAACGTG	Eurofins	Custom-made
Human/Mouse *TWIST*<br>Rev: CAGCTTGCCATCTTGGAGTC	Eurofins	Custom-made
Mouse *E-cadherin*<br>Fw: GACTGTGAAGGGACGGTCAAC	Eurofins	Custom-made

Mouse *E-cadherin*<br>Rev: CCACCGTTCTCCTCCGTAGA	Eurofins	Custom-made
Mouse *Fibronectin*<br>Fw: CCCAGACTTATGGTGGCAATTC	Eurofins	Custom-made
Mouse *Fibronectin*<br>Rev: AATTTCCGCCTCGAGTCTGA	Eurofins	Custom-made
Mouse *Gapdh*<br>Fw: TGTGTCCGTCGTGGATCTGA	Eurofins	Custom-made
Mouse *Gapdh*<br>Rev: CCTGCTTCACCACCTTCTTGA	Eurofins	Custom-made
Mouse *N-cadherin*<br>Fw: AGGCGGAGACCTGTGAAACTC	Eurofins	Custom-made
Mouse *N-cadherin*<br>Rev: CCATTAAGCCGGTTGATGGT	Eurofins	Custom-made
Mouse *Slug*<br>Fw: TGTGTCTGCAAGATCTGTGGC	Eurofins	Custom-made
Mouse *Slug*<br>Rev: TCCCCAGTGTGAGTTCTAATGTG	Eurofins	Custom-made
Mouse *Snail*<br>Fw: CCACTGCAACCGTGCTTTT	Eurofins	Custom-made
Mouse *Snail*<br>Rev: CAGATCCGAGTGGGTTTGG	Eurofins	Custom-made
Mouse *Sox2*<br>Fw: CACAACTCGGAGATCAGCAA	Eurofins	Custom-made
Mouse *Sox2*<br>Rev: TCTCGGTCTCGGACAAAAGT	Eurofins	Custom-made
Mouse *Tgfβ1*<br>Fw: CGATCGCTACCCGGCGTTCC	Eurofins	Custom-made
Mouse *Tgfβ1*<br>Rev: GGCGTATCAGTGGGGGTCA	Eurofins	Custom-made
Mouse *Vimentin*<br>Fw: AGACCAGAGATGGACAGGTGA	Eurofins	Custom-made
Mouse *Vimentin*<br>Rev: CTGGTACTGCACTGTTGCAC	Eurofins	Custom-made
Mouse *Zeb1*<br>Fw: TTCTGCAGCAACAAGACACC	Eurofins	Custom-made
Mouse *Zeb1*<br>Rev: TCATCATGACTGXTGGCTTC	Eurofins	Custom-made
Mouse *Zeb2*<br>Fw: CGACACGGCCATTATTTACC	Eurofins	Custom-made
Mouse *Zeb2*<br>Rev: GGCAAAAGCATCTGGAGTTC	Eurofins	Custom-made
Oligonucleotides for cloning		
AseI-mouse *E-cadherin* promoter<br>Fw: AAAAAATTAATCCCGGGAGGTAC<br>C GAGCTCTTACGCGTGCTAG	Eurofins	Custom-made
Mouse *E-cadherin* promoter-*AgeI*<br>Rev: TTTTTACCGGTGCGGGTGCGGT<br>CGGGCAGGGCCGG	Eurofins	Custom-made

AseI-mouse *ESRP2* promoter<br>Fw: AAAAAATTAATAAGACCGATAGGA<br>AGCAGCCCGCTTTTCCAAACA	Eurofins	Custom-made
Mouse *ESRP2* promoter-*AgeI*<br>Rev: TTTTTACCGGTAGAGGAAGGGG<br>GCGCTCGGCCACACACG	Eurofins	Custom-made
AseI-human *miR200s* promoter<br>Fw: AAAAAATTAATGGTACCGAGCTCT<br>TACGCGTGCTAGCAGAGGTG	Eurofins	Custom-made
Human *miR200s* promoter-*AgeI*<br>Rev: TTTTTACCGGTAAGCTTACTTAG<br>ATCGCAGATCTCCTGGCACAGG	Eurofins	Custom-made

Table S5. List of oligonucleotides used.

Reagent	Source	Specification
anti-β-actin (AC-15)<br>(1:1,000 immunoblot)	Santa Cruz Biotechnology Inc.	Cat# sc-69879<br>RRID:AB_1119529
Anti-CD31 (1:100 immunohistochemistry)	Cell Signaling Technology	Cat# 77699,<br>RRID:AB_2722705
anti-CD44<br>(1:1,000 immunoblot)	Abcam	Cat# ab157107<br>RRID:AB_2847859
anit-CD63<br>(1:1,000 immunoblot)	BioLegend	Cat# 143901<br>RRID:AB_11203908
anti-CD81<br>(1:200 immunoblot)	Santa Cruz Biotechnology, Inc.	Cat# sc-166029<br>RRID:AB_2275892
anti-Cytochrome C<br>(1:500 immunoblot)	BD Biosciences-Europe	Cat# 556433<br>RRID:AB_396417
anti-DsRed<br>(1:1,000 immunoblot)	Clontech Laboratories Inc.	Cat# 632393
anti-RFP/DsRed<br>(1:100 immunohistochemistry)	MBL International Corp.	PM005<br>RRID:AB_591279
anti-E-cadherin (24E10)<br>(1:100 immunofluorescence)	Cell Signaling Technology	Cat# 3195<br>RRID:AB_2291471
anti-E-cadherin<br>(1:20,000 immunoblot; 1:100 immunohistochemistry)	BD Biosciences-Europe	Cat# 610182<br>RRID:AB_397581
anti-EpCAM<br>(1:1,000 immunoblot)	Cell Signaling Technology	Cat# 2929<br>RRID:AB_2098657
anti-Fibronectin<br>(1:30,000 immunoblot; 1:1,000 immunofluorescence)	Sigma-Aldrich AB	Cat# F3648,<br>RRID:AB_476976
anti-Id1 (Z-8)<br>(1:1,000 immunoblot)	Santa Cruz Biotechnology Inc.	Cat# sc-427<br>RRID:AB_631700
anti-Ki-67<br>(1:400 immunohistochemistry)	Abcam	ab15580<br>RRID:AB_443209
anti-N-Cadherin<br>(1:50,0000 immunoblot; 1:100 immunofluorescence)	BD Biosciences-Europe	Cat# 610920,<br>RRID:AB_2077527
anti-Smad1<br>(1:1,000 immunoblot)	Abcam	Cat# ab33902,<br>RRID:AB_777975
anti-Smad2/3<br>(1:1,000 immunoblot)	BD Biosciences-Europe	Cat# 610843,<br>RRID:AB_39816
anti-Snail (C15D3)<br>(1:1,000 immunoblot)	Cell Signaling Technology	Cat# 3879<br>RRID:AB_2255011

anti-p-Smad1/Ser463/465)/ Smad5(463/465)/Smad8(Ser465/467) (1:1,000 immunoblot)	Cell Signaling Technology	Cat# 9511 RRID:AB_331671
anti-p-Smad2 (1:1,000 immunoblot)	Ludwig Institute for Cancer Research, Uppsala, Sweden	Peter ten Dijke (author)
anti-p-Smad2 (1:200 immunofluorescence)	Thermo Fisher Scientific	Cat#44-244G RRID: AB_2533614
anti-Sox2 (1:1,000 immunoblot)	Merck Millipore	Cat# AB5603 RRID:AB_2286686
anti-Sox2 (1:100 immunohistochemistry)	Abcam	Cat# ab79351 RRID:AB_10710406
anti-Vimentin (D21H3) (1:100 immunoblot, immunofluorescence, immunohistochemistry)	Cell Signaling Technology	Cat# 5741 RRID:AB_10695459
anti-ZEB1 (1:1,000 immunoblot)	Sigma-Aldrich AB	Cat# HPA027524 RRID:AB_1844977
anti-ZO-1 (1:100 immunofluorescence)	Invitrogen	Cat# 33-9100 RRID:AB_87181
Alexa647-anti-CD106 (1:100 flow)	BD Biosciences-Europe	Cat# 561612 RRID:AB_10896662
APC-anti-CD24 (1:100 flow)	BD Biosciences-Europe	Cat# 562349 RRID:AB_11151896
BB515-anti-EpCAM (1:100 flow)	BD Biosciences-Europe	Cat# 565425 RRID:AB_2739232
BB515-Streptavidin (1:100 flow)	BD Biosciences-Europe	Cat# 564453 RRID:AB_2869580
Biotin-anti-CD51 (1:100 flow)	BioLegend	Cat# 104103 RRID:AB_313072
Biotin- anti-CD104 (1:100 flow)	BioLegend	Cat# 123603 RRID:AB_961034
BV510-Streptavidin (1:100 flow)	BioLegend	Cat# 405233
BV605-anti-CD44 (1:100 flow)	BD Biosciences-Europe	Cat# 563058 RRID:AB_2737979
BV605-anti-CD61 (1:100 flow)	BD Biosciences-Europe	Cat# 740348 RRID:AB_2740081
anti-Rabbit IgG (H+L) Secondary, HRP	Thermo Fisher Scientific	Cat# 65-6120 RRID:AB_2533967

anti-Mouse IgG (H+L) Secondary, HRP	Thermo Fisher Scientific	Cat# 62-6520 RRID:AB_88369
anti-mouse Alexa 488 Fluor	Thermo Fisher Scientific	Cat# A21202 RRID:AB_141607
anti-rabbit Alexa 488 Fluor	Thermo Fisher Scientific	Cat# A21206 RRID:AB_2535792

Table S6. List of antibodies used with dilution factors and application (immunoblot, immunofluorescence, immunohistochemistry or flow cytometry (flow).

Table S7. Excel file with the list of differentially expressed genes in cells, related to the data of Fig. S4. Excel table with 9 sheets labelled as follows and containing the following data:

Cells Control vs EMT: Gene expression values for the 3 control and 3 EMT cellular samples with associated fold-changes and p-values.

Cells Control-EMT 2>log2: Genes up-regulated (logFC>2) in 3 EMT cellular samples relative to control cells.

Cells Control-EMT -1<log2: Genes down-regulated (logFC>2) in 3 EMT cellular samples relative to control cells.

Cells Control vs MET: Gene expression values for the 3 control and 3 MET cellular samples with associated fold-changes and p-values.

Cells Control-MET 2>log2: Genes up-regulated (logFC>2) in 3 MET cellular samples relative to control cells.

Cells Control-MET -1<log2: Genes down-regulated (logFC>2) in 3 MET cellular samples relative to control cells.

Cells EMT vs MET: Gene expression values for the 3 EMT and 3 MET cellular samples with associated fold-changes and p-values.

Cells EMT-MET 2>log2: Genes up-regulated (logFC>2) in 3 EMT cellular samples relative to MET cells.

Cells EMT-MET -1<log2: Genes down-regulated (logFC>2) in 3 EMT cellular samples relative to MET cells.

Table S8. Excel file (FC_Cell_FDR) with the list of differentially expressed genes in cells, related to the data of Fig. S4B. Excel table with 3 sheets labelled as *CtrVs.EMT*,

CtrVs.MET and *METvsEMT*, showing logFC (fold-change), logCPM, p-values and FDR (false discovery rate).

Table S9. Excel file (EMT_GSEA_h.all.v7.1.entrez.gmt_EMT) with the GSEA data of differentially expressed cellular RNAs from EMT versus control cells, related to the data of Fig. S4C. Excel table labelled as follows and containing the following data:
GSEA_h.all.v7.1.entrez.gmt_EMT: GSEA analyzing 39 biological pathways with associated genes per pathway listed on the last column.

Table S10. Excel file (METvsEMT_GSEA_h.all.v7.1.entrez.gmt_EMT) with the GSEA data of differentially expressed cellular RNAs from MET vs EMT cells, related to the data of Fig. S4D. Excel table labelled as follows and containing the following data:
GSEA_h.all.v7.1.entrez.gmt_EMTv: GSEA analyzing 32 biological pathways with associated genes per pathway listed on the last column.

Table S11. Excel file (KEGG_CellSimilar_Down) with the GO analysis data of differentially down-regulated cellular RNAs from EMT cells, related to the data of Fig. S4F, G. Excel table labelled as follows and containing the following data:
KEGG_CellSimilar_Down: GO analyzing 12 biological pathways with associated genes per pathway from the KEGG database.

Supporting figures

Figure S1

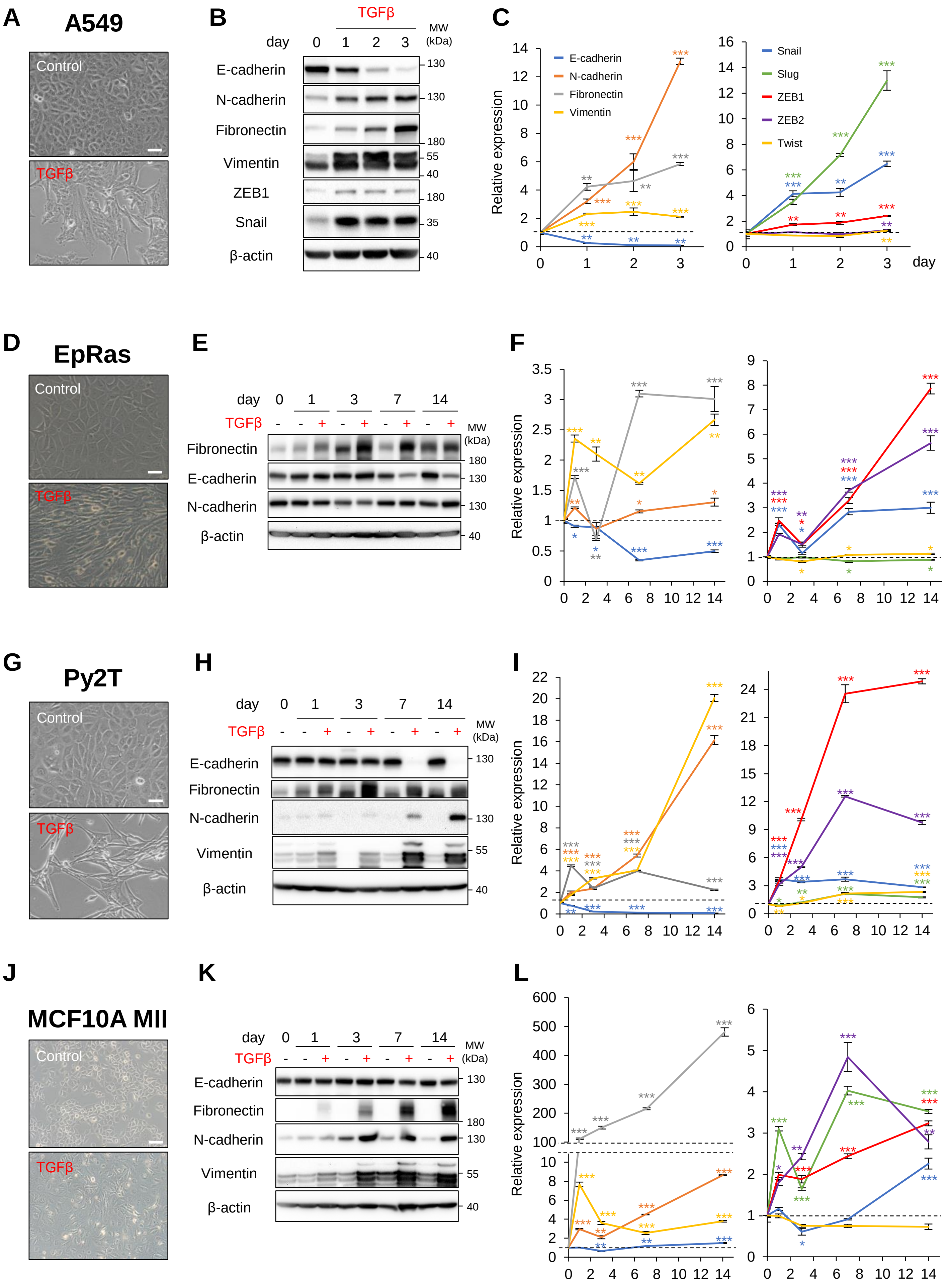

A
A549
Control
TGFβ
B
TGFβ
day 0 1 2 3
MW (kDa)
E-cadherin
N-cadherin
Fibronectin
Vimentin
ZEB1
Snail
β-actin
130
130
180
55
40
180
35
40
C
Relative expression
E-cadherin
N-cadherin
Fibronectin
Vimentin
Snail
Slug
ZEB1
ZEB2
Twist
day
D
EpRas
Control
TGFβ
E
day 0 1 3 7 14
TGFβ
Fibronectin
E-cadherin
N-cadherin
β-actin
MW (kDa)
180
130
130
40
F
Relative expression
G
Py2T
Control
TGFβ
H
day 0 1 3 7 14
TGFβ
E-cadherin
Fibronectin
N-cadherin
Vimentin
β-actin
MW (kDa)
130
130
55
40
I
Relative expression
J
MCF10A MII
Control
TGFβ
K
day 0 1 3 7 14
TGFβ
E-cadherin
Fibronectin
N-cadherin
Vimentin
β-actin
MW (kDa)
130
180
130
55
40
L
Relative expression

Fig. S1. EMT induction in candidate cancer cell lines**.** (A, D, G, J) Representative DIC images of the indicated cell lines treated with 5 ng/ml TGFβ1 or vehicle (control) (n=3 independent experiments). Scale bar, 50 μm. (B, E, H, K) Immunoblot of A549 (B), EpRas (H), Py2T (H) and MCF10A MII (K) cells treated with 5 ng/ml TGFβ1 (+) or vehicle control (-) for the indicated time periods analyzing epithelial and mesenchymal proteins, and β-actin as a loading control, along with molecular size markers (n=3 independent experiments). For original images, see Fig. S11. (C, F, I, L) qRT–PCR of relative expression of epithelial and mesenchymal genes in A549 (C), EpRas (F), Py2T (I) and MCF10A MII (L) cells treated as in panels B, E, H, K, respectively. The expression levels were normalized to *GAPDH*/*Gapdh*. Horizontal dotted lines indicate relative expression equal to 1 (in panel L, the y-axis scale is broken by double dotted lines to save space). Average values and SD from n=3 biological replicates, each with technical triplicates, and p-values (*p<0.05, **p<0.01, ***p<0.001) after two-tailed paired Student's t-test.

Figure S2

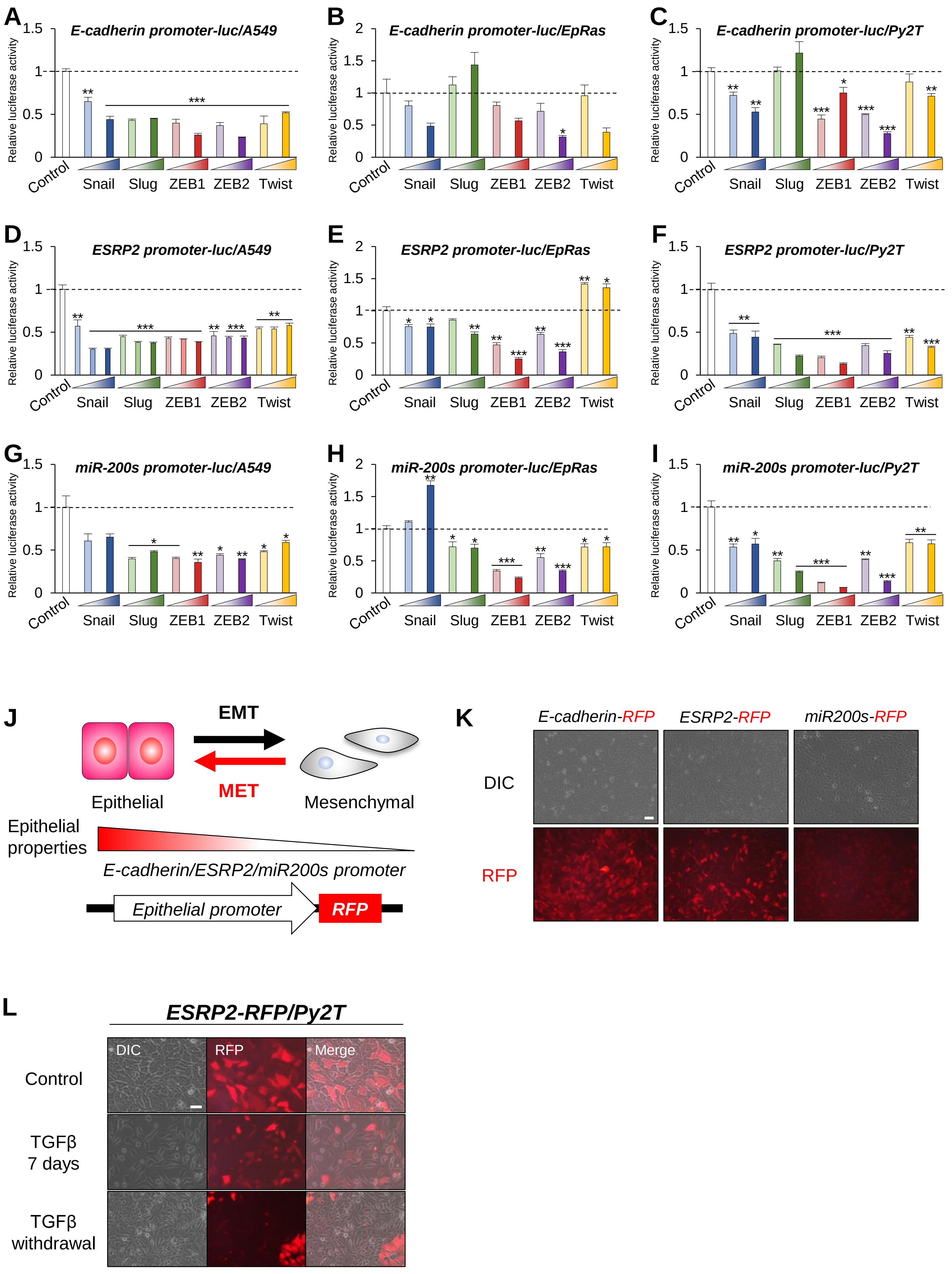
A
E-cadherin promoter-luc/A549
B
E-cadherin promoter-luc/EpRas
C
E-cadherin promoter-luc/Py2T
D
ESRP2 promoter-luc/A549
E
ESRP2 promoter-luc/EpRas
F
ESRP2 promoter-luc/Py2T
G
miR-200s promoter-luc/A549
H
miR-200s promoter-luc/EpRas
I
miR-200s promoter-luc/Py2T
Relative luciferase activity
Control
Snail
Slug
ZEB1
ZEB2
Twist
J
EMT
MET
Epithelial
Mesenchymal
Epithelial properties
E-cadherin/ESRP2/miR200s promoter
Epithelial promoter
RFP
K
E-cadherin-RFP
ESRP2-RFP
miR200s-RFP
DIC
RFP
L
ESRP2-RFP/Py2T
DIC
RFP
Merge
Control
TGFβ 7 days
TGFβ withdrawal

Fig. S2. Screening of epithelial promoters in cancer cell lines. (A-I) Epithelial gene promoter activities (*E-cadherin* (A-C), *Esrp2* (D-F), *miR-200s* (G-I)) were analyzed by luciferase reporter assay after transient transfection of two or three doses of the indicated EMT-TFs. Firefly luciferase activities were normalized to renilla luciferase activity. Average values and SD from n=3 biological replicates, each with technical triplicates, and p-values (*<0.05, **p<0.01, ***p<0.001) after two-tailed paired Student's t-test. Horizontal dotted lines indicate relative expression equal to 1. (J) Scheme of EMT imaging probe, which consists of an epithelial gene promoter (*E-cadherin*, *Esrp2* or *miR-200s*) driving expression of RFP. (K) DIC and RFP imaging of Py2T cells stably expressing *E-cadherin*-, *Esrp2*- or *miR-200s*-driven RFP (n=3 independent experiments). (L) Representative imaging of DIC and RFP during EMT (TGFβ) and MET (TGFβ withdrawal) in *Esrp2*-RFP/Py2T cells (n=3 independent experiments). Scale bars (K, L), 50 µm.

Figure S3

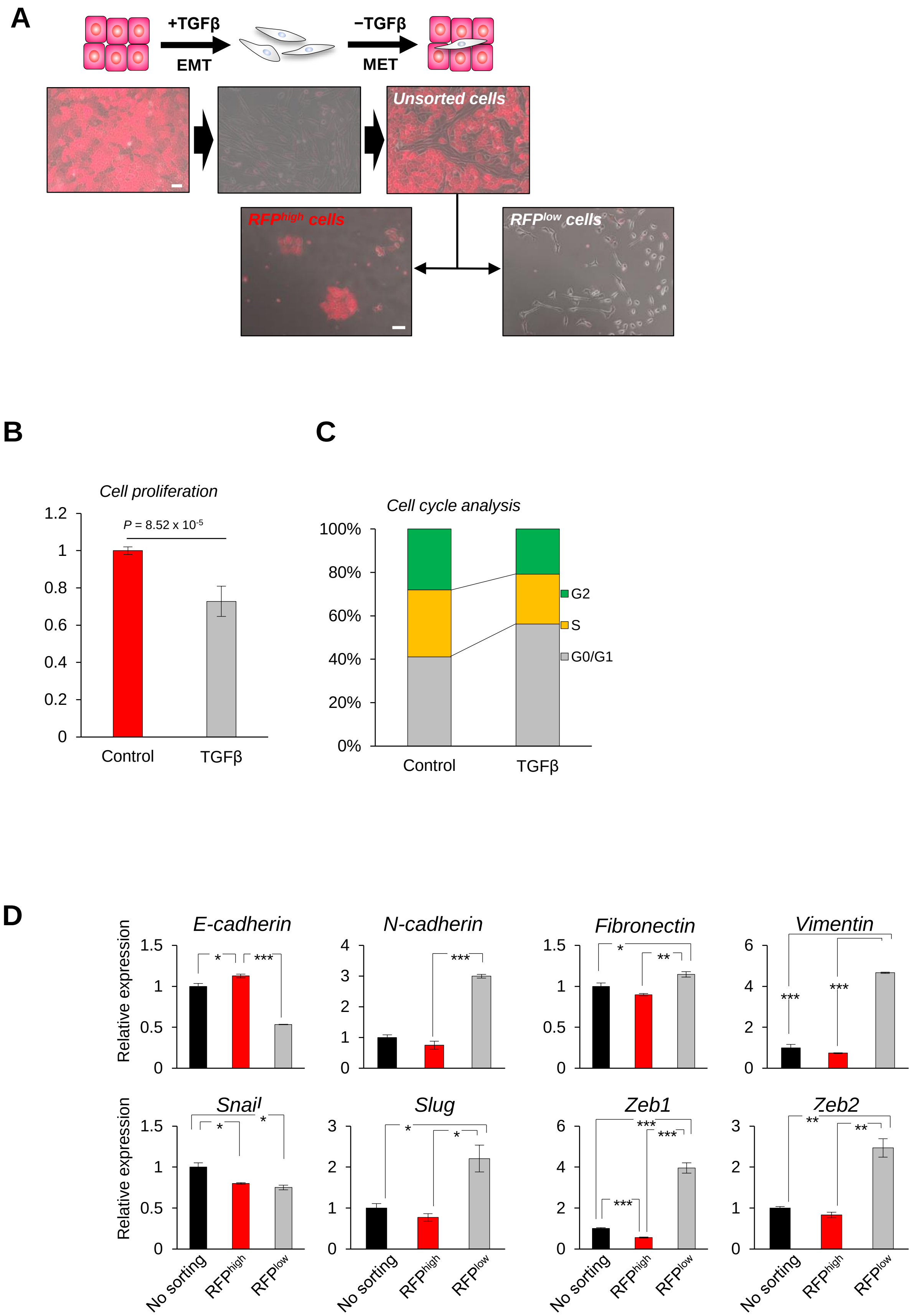
A
+TGFβ
EMT
−TGFβ
MET
Unsorted cells
RFPhigh cells
RFPlow cells
B
Cell proliferation
P = 8.52 x 10-5
1.2
1
0.8
0.6
0.4
0.2
0
Control
TGFβ
C
Cell cycle analysis
100%
80%
60%
40%
20%
0%
G2
S
G0/G1
Control
TGFβ
D
E-cadherin
N-cadherin
Fibronectin
Vimentin
Snail
Slug
Zeb1
Zeb2
Relative expression
No sorting
RFPhigh
RFPlow

Fig. S3. RFP^{high} cells express epithelial and RFP^{low} cells express mesenchymal genes. (A) Schematic overview of the RFP^{high} or RFP^{low} cell sorting experiments. DIC/RFP fluorescence overlay images of *E-cadherin*-RFP/Py2T cells during EMT/MET. Scale bar, 50 µm. (B) *E-cadherin*-RFP/Py2T cell proliferation analysis without (control) or with 5 ng/ml TGFβ1 stimulation for 7 days by MTS assay; control cells are normalized to 1. Average values and SD from n=3 biological replicates, each with technical triplicates, and p-values (p-value) after two-tailed paired Student's t-test. (C) Cell cycle analysis by flow cytometry expressed as % of cells in each cell cycle phase (biological conditions as in panel B). (D) Relative expression of epithelial and mesenchymal genes in unsorted, RFP^{high} and RFP^{low} cells. Average values and SD from n=3 biological replicates, each with technical triplicates, and p-values (*<0.05, **p<0.01, ***p<0.001) after two-tailed paired Student's t-test.

Figure S4

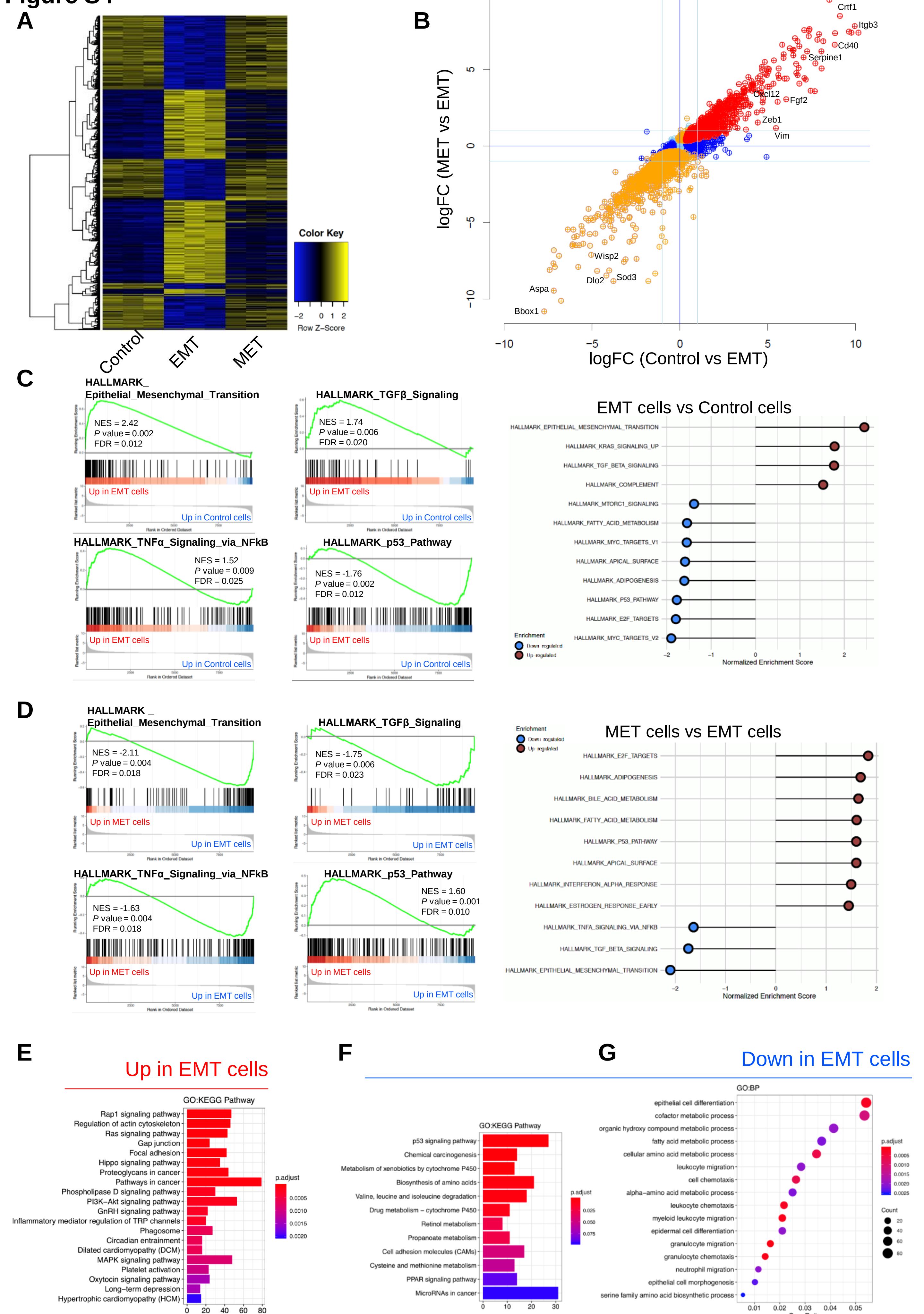

A
Color Key
Row Z-Score
Control
EMT
MET
B
logFC (MET vs EMT)
logFC (Control vs EMT)
Crtf1
Itgb3
Cd40
Serpine1
Cxcl12
Fgf2
Zeb1
Vim
Wisp2
Dlo2
Sod3
Aspa
Bbox1
C
HALLMARK_Epithelial_Mesenchymal_Transition
NES = 2.42
P value = 0.002
FDR = 0.012
HALLMARK_TGFβ_Signaling
NES = 1.74
P value = 0.006
FDR = 0.020
HALLMARK_TNFα_Signaling_via_NFkB
NES = 1.52
P value = 0.009
FDR = 0.025
HALLMARK_p53_Pathway
NES = -1.76
P value = 0.002
FDR = 0.012
Up in EMT cells
Up in Control cells
EMT cells vs Control cells
HALLMARK_EPITHELIAL_MESENCHYMAL_TRANSITION
HALLMARK_KRAS_SIGNALING_UP
HALLMARK_TGF_BETA_SIGNALING
HALLMARK_COMPLEMENT
HALLMARK_MTORC1_SIGNALING
HALLMARK_FATTY_ACID_METABOLISM
HALLMARK_MYC_TARGETS_V1
HALLMARK_APICAL_SURFACE
HALLMARK_ADIPOGENESIS
HALLMARK_P53_PATHWAY
HALLMARK_E2F_TARGETS
HALLMARK_MYC_TARGETS_V2
Enrichment
Down regulated
Up regulated
Normalized Enrichment Score
D
HALLMARK _Epithelial_Mesenchymal_Transition
NES = -2.11
P value = 0.004
FDR = 0.018
HALLMARK_TGFβ_Signaling
NES = -1.75
P value = 0.006
FDR = 0.023
HALLMARK_TNFα_Signaling_via_NFkB
NES = -1.63
P value = 0.004
FDR = 0.018
HALLMARK_p53_Pathway
NES = 1.60
P value = 0.001
FDR = 0.010
Up in MET cells
Up in EMT cells
MET cells vs EMT cells
HALLMARK_E2F_TARGETS
HALLMARK_ADIPOGENESIS
HALLMARK_BILE_ACID_METABOLISM
HALLMARK_FATTY_ACID_METABOLISM
HALLMARK_P53_PATHWAY
HALLMARK_APICAL_SURFACE
HALLMARK_INTERFERON_ALPHA_RESPONSE
HALLMARK_ESTROGEN_RESPONSE_EARLY
HALLMARK_TNFA_SIGNALING_VIA_NFKB
HALLMARK_TGF_BETA_SIGNALING
HALLMARK_EPITHELIAL_MESENCHYMAL_TRANSITION
E
Up in EMT cells
GO:KEGG Pathway
Rap1 signaling pathway
Regulation of actin cytoskeleton
Ras signaling pathway
Gap junction
Focal adhesion
Hippo signaling pathway
Proteoglycans in cancer
Pathways in cancer
Phospholipase D signaling pathway
PI3K–Akt signaling pathway
GnRH signaling pathway
Inflammatory mediator regulation of TRP channels
Phagosome
Circadian entrainment
Dilated cardiomyopathy (DCM)
MAPK signaling pathway
Platelet activation
Oxytocin signaling pathway
Long–term depression
Hypertrophic cardiomyopathy (HCM)
p.adjust
F
GO:KEGG Pathway
p53 signaling pathway
Chemical carcinogenesis
Metabolism of xenobiotics by cytochrome P450
Biosynthesis of amino acids
Valine, leucine and isoleucine degradation
Drug metabolism – cytochrome P450
Retinol metabolism
Propanoate metabolism
Cell adhesion molecules (CAMs)
Cysteine and methionine metabolism
PPAR signaling pathway
MicroRNAs in cancer
p.adjust
G
Down in EMT cells
GO:BP
epithelial cell differentiation
cofactor metabolic process
organic hydroxy compound metabolic process
fatty acid metabolic process
cellular amino acid metabolic process
leukocyte migration
cell chemotaxis
alpha–amino acid metabolic process
leukocyte chemotaxis
myeloid leukocyte migration
epidermal cell differentiation
granulocyte migration
granulocyte chemotaxis
neutrophil migration
epithelial cell morphogenesis
serine family amino acid biosynthetic process
p.adjust
Count

Fig. S4. Transcriptome of the EMT and MET cell states. (A) Heat-map and hierarchical clustering across the three biological conditions indicated for the false discovery rate (FDR)<0.001. Color key for gene expression is included. (B) Scatter plot of cellular RNAs for the comparison between EMT vs control and EMT vs MET. Individual genes (dots) are colored as red (up-regulated genes in EMT cells only and down-regulated in both control and MET cells), blue (not differentially regulated genes) and orange (down-regulated genes in EMT cells only and up-regulated in both control and MET cells). Dotted lines mark the logFC values of -1 and +1 on each axis. (C, D) GSEA for cellular RNAs analyzed in the biological conditions of EMT relative to control (C) and in MET cells relative to EMT (D). GSEA graphs are shown on the left side with deconvolution of the up- and down-regulated gene sets listed on the right side. Statistical analysis performed using the GSEA algorithm with NES, normalized enrichment score; p-value and FDR. (E-G) Gene Ontology (GO), biological process (BP), KEGG and pathway-enrichment analysis for the genes up-regulated in EMT cells (E), and for the genes down-regulated in EMT cells (F, G). Adjusted p-value is color-coded and gene number per GO term is visualized based on the surface size of the circle.

Figure S5

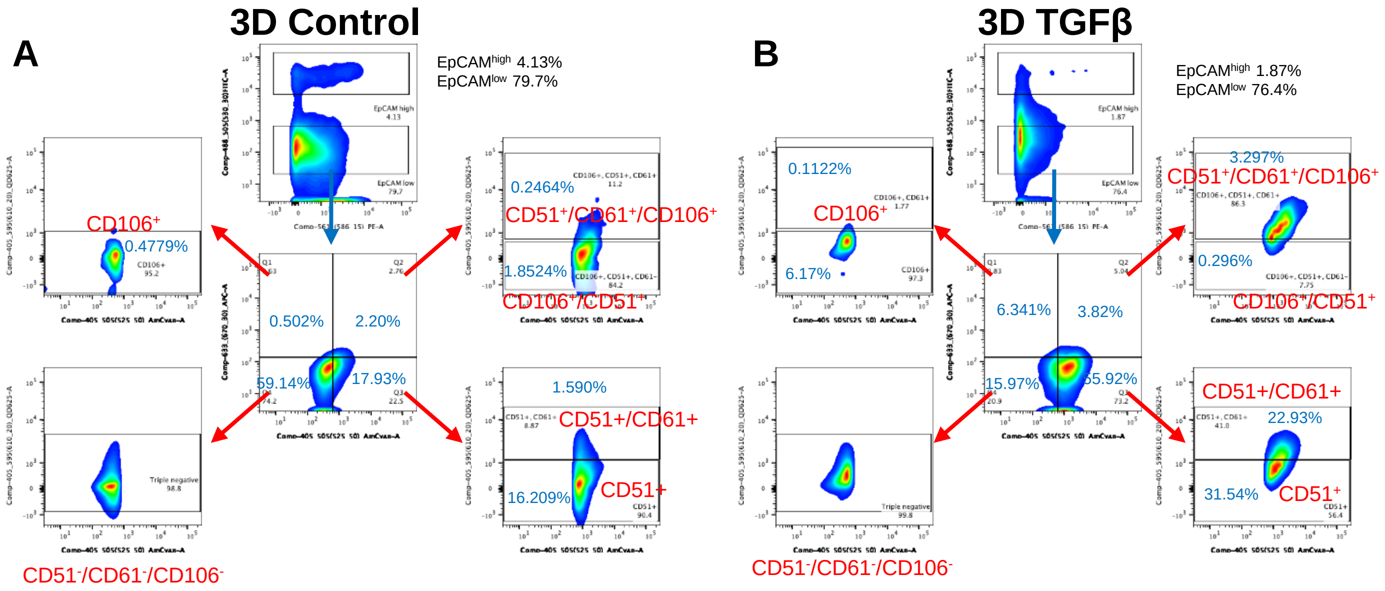

3D Control
A
EpCAM^high 4.13%
EpCAM^low 79.7%
EpCAM high 4.13
EpCAM low 79.7
CD106+
0.4779%
CD106+ 95.2
0.502%
2.20%
59.14%
17.93%
0.2464%
CD106+, CD51+, CD61+ 11.2
CD51+/CD61+/CD106+
1.8524%
CD106+, CD51+, CD61- 84.2
CD106+/CD51+
Triple negative 98.8
CD51-/CD61-/CD106-
1.590%
CD51+, CD61+ 8.87
CD51+/CD61+
16.209%
CD51+
CD51+ 90.4
3D TGFβ
B
EpCAM^high 1.87%
EpCAM^low 76.4%
EpCAM high 1.87
EpCAM low 76.4
0.1122%
CD106+, CD61+ 1.77
CD106+
6.17%
CD106+ 97.3
6.341%
3.82%
15.97%
55.92%
3.297%
CD51+/CD61+/CD106+
CD106+, CD51+, CD61+ 86.3
0.296%
CD106+, CD51+, CD61- 7.75
CD106+/CD51+
Triple negative 99.8
CD51-/CD61-/CD106-
CD51+/CD61+
CD51+, CD61+ 41.0
22.93%
31.54%
CD51+
CD51+ 56.4

Fig. S5. EMT-score analysis by flow cytometry after TGFβ stimulation in 2D and 3D cultures. Flow cytometry analysis of *E-cadherin*-RFP/Py2T cells in 3D (A, B) cultures unstimulated (control, A) or stimulated with 5 ng/ml TGFβ1 for 7 days (B). The top plots show EpCAM expression; the bottom central plots (blue arrows) show partial EMT marker analysis in EpCAM^{low} cells. Each of the 4 quadrants of the partial EMT analysis is shown (red arrows) around the central bottom plots. Three biological replicates were used for the analysis.

Figure S6

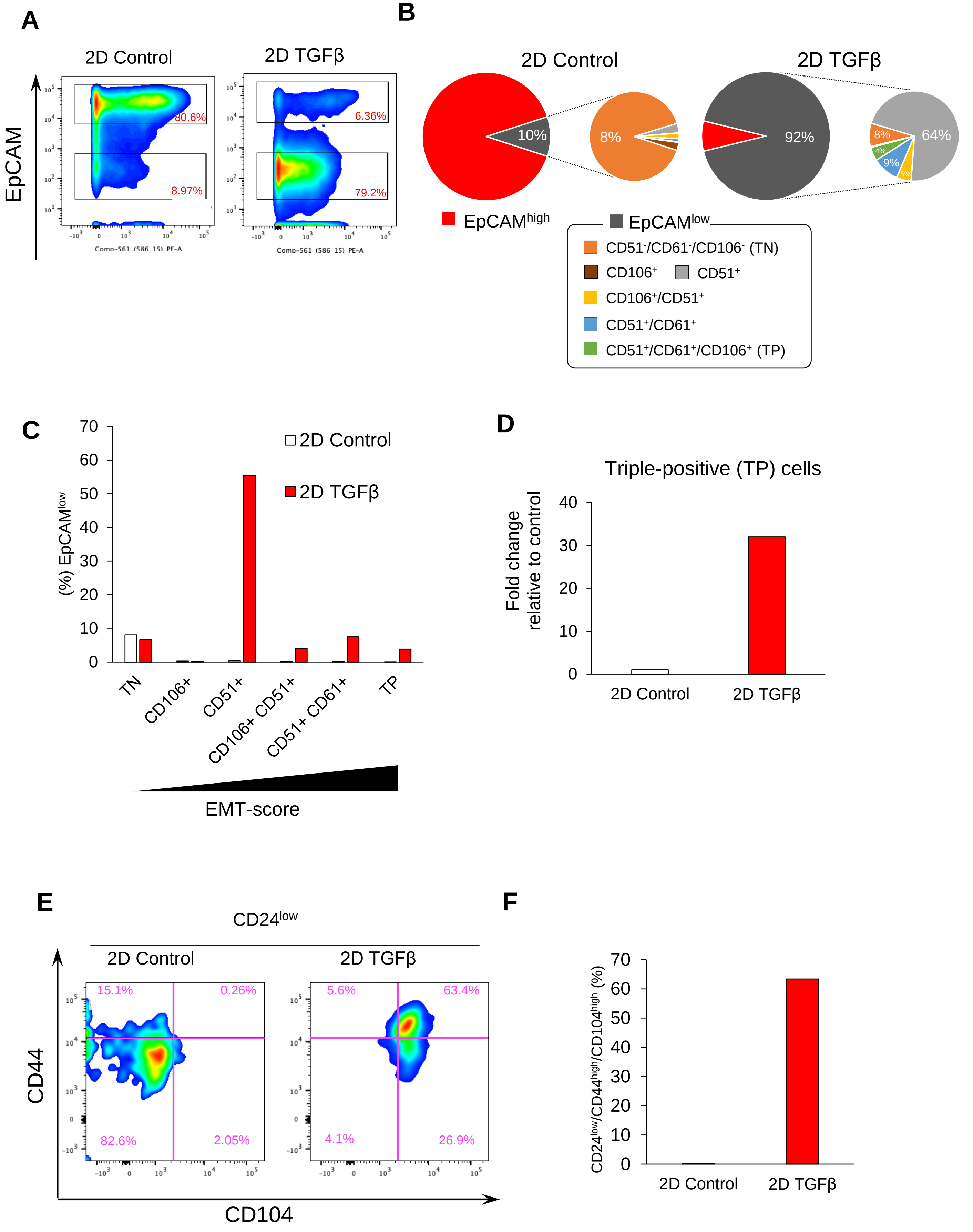
A
2D Control
2D TGFβ
EpCAM
80.6%
8.97%
6.36%
79.2%
Comp-561 (586 15) PE-A
B
2D Control
2D TGFβ
10%
8%
92%
64%
8%
4%
9%
5%
EpCAMhigh
EpCAMlow
CD51-/CD61-/CD106- (TN)
CD106+
CD51+
CD106+/CD51+
CD51+/CD61+
CD51+/CD61+/CD106+ (TP)
C
2D Control
2D TGFβ
(%) EpCAMlow
TN
CD106+
CD51+
CD106+ CD51+
CD51+ CD61+
TP
EMT-score
D
Triple-positive (TP) cells
Fold change relative to control
2D Control
2D TGFβ
E
CD24low
2D Control
2D TGFβ
CD44
CD104
15.1%
0.26%
82.6%
2.05%
5.6%
63.4%
4.1%
26.9%
F
CD24low/CD44high/CD104high (%)
2D Control
2D TGFβ

Fig. S6. Partial EMT, $EpCAM^{low}/CD51^{+}$ or $CD24^{low}/CD44^{high}/CD104^{high}$ cells, are preferentially induced by TGFβ under 2D culture conditions**.** (A) EMT-score analysis by flow cytometry in the indicated two biological conditions and with detailed analysis in Fig. S7. $EpCAM^{high}$ or $EpCAM^{low}$ populations were gated. Three biological replicates were used for the analysis. (B) Pie charts illustrating the indicated color-coded cell populations as percentage of the total under the two biological conditions used. The data are identical to those of panel A (n=3 biological replicates). The cell type analysis was performed by flow cytometry of the indicated surface proteins in the $EpCAM^{low}$ population. Detailed analysis is shown in Fig. S7. (C) Impact of TGFβ on EMT-scores under 2D culture conditions. The data are identical to those of panel B (n=3 biological replicates). Triple-negative (TN) cells represent early EMT and correspond to $EpCAM^{low}/CD51^{-}/CD61^{-}/CD106^{-}$ and triple-positive (TP) cells represent complete EMT and correspond to $EpCAM^{low}/CD51^{+}/CD61^{+}/CD106^{+}$. (D) The $EpCAM^{low}$/triple-positive cell population generated after TGFβ stimulation under 2D conditions is graphed relative to the control (no TGFβ) condition, which is normalized to 1. (E) Partial EMT-score analysis by flow cytometry in the indicated four biological conditions and with detailed analysis in Fig. S7C. $CD24^{low}$ cells were gated first, and CD44 and CD104 cell surface expression was analyzed using the indicated fluorescently-conjugated antibodies. Three biological replicates were used for the analysis. (F) The percent of $CD24^{low}/CD44^{high}/CD104^{high}$ cells is graphed for cells responding to TGFβ under 2D culture conditions.

Figure S7

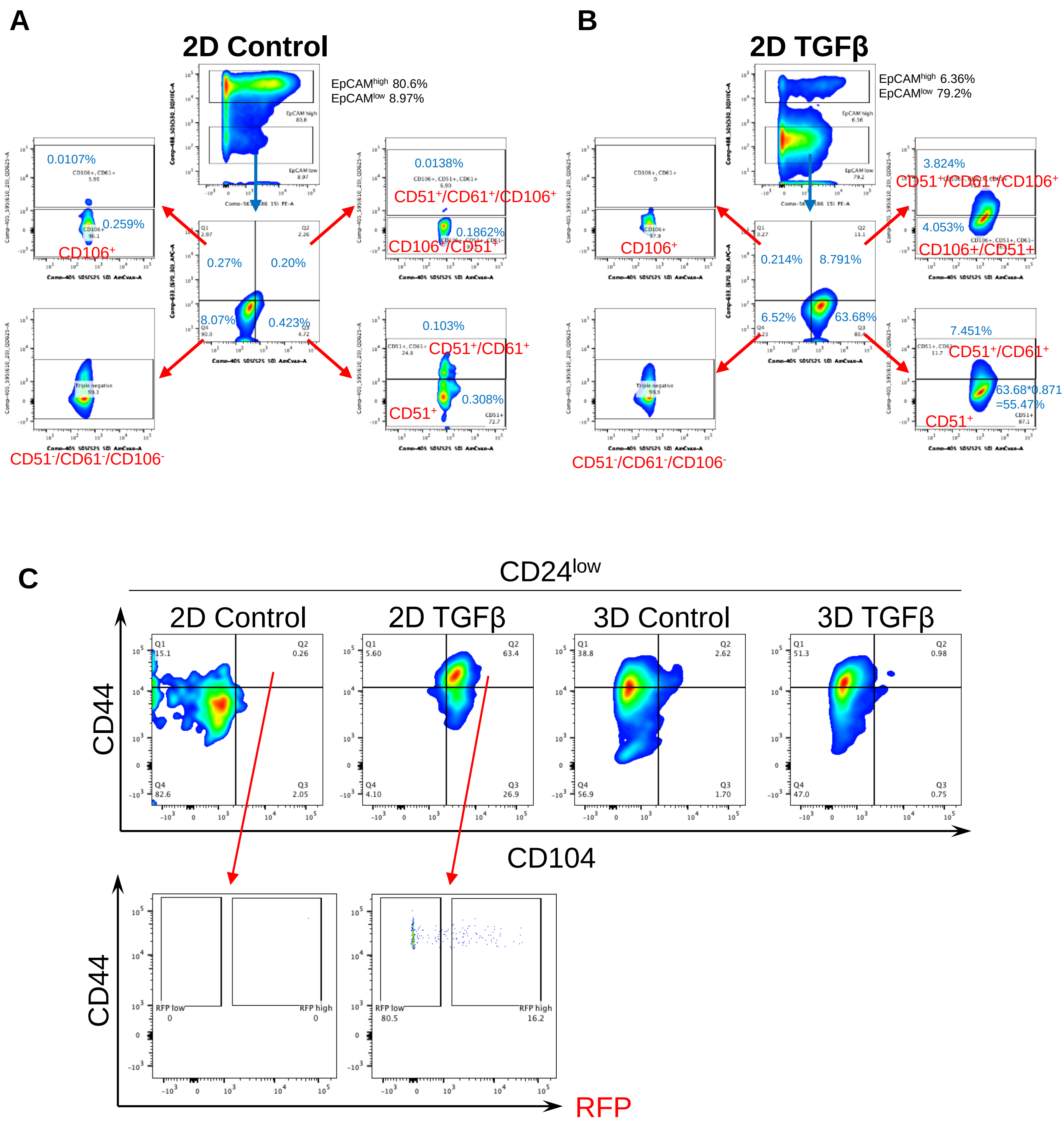

A
2D Control
EpCAMhigh 80.6%
EpCAMlow 8.97%
EpCAM high 80.6
EpCAM low 8.97
0.0107%
CD106+, CD61+ 3.95
0.259%
CD106+
0.27%
0.20%
8.07%
0.423%
0.0138%
CD106+, CD51+, CD61+ 6.90
CD51+/CD61+/CD106+
0.1862%
CD106+/CD51+
0.103%
CD51+, CD61+ 24.8
CD51+/CD61+
0.308%
CD51+
CD51+ 72.7
Triple negative 99.3
CD51-/CD61-/CD106-
B
2D TGFβ
EpCAMhigh 6.36%
EpCAMlow 79.2%
EpCAM high 6.36
EpCAM low 79.2
CD106+, CD61+ 0
CD106+ 97.9
CD106+
0.214%
8.791%
6.52%
63.68%
3.824%
CD51+/CD61+/CD106+
4.053%
CD106+/CD51+
7.451%
CD51+/CD61+
63.68*0.871 =55.47%
CD51+
CD51+ 87.1
Triple negative 99.9
CD51-/CD61-/CD106-
C
CD24low
2D Control
2D TGFβ
3D Control
3D TGFβ
Q1 15.1
Q2 0.26
Q4 82.6
Q3 2.05
Q1 5.60
Q2 63.4
Q4 4.10
Q3 26.9
Q1 38.8
Q2 2.62
Q4 56.9
Q3 1.70
Q1 51.3
Q2 0.98
Q4 47.0
Q3 0.75
CD44
CD104
RFP low 0
RFP high 0
RFP low 80.5
RFP high 16.2
RFP

Fig. S7. EMT-score analysis by flow cytometry after TGFβ stimulation in 2D cultures. (A, B) Flow cytometry analysis of *E-cadherin*-RFP/Py2T cells in 2D cultures unstimulated (control, A) or stimulated with 5 ng/ml TGFβ1 for 7 days (B). The top plots show EpCAM expression; the bottom central plots (blue arrows) show partial EMT marker analysis in $EpCAM^{low}$ cells. Each of the 4 quadrants of the partial EMT analysis is shown (red arrows) around the central bottom plots. (C) Flow cytometry analysis of cells cultured as in panels A, B. After selection of the $CD24^{low}$ cell population, CD44 and CD104 expression was analyzed (top 4 plots). The $CD44^{high}/CD104^{high}$ cell populations (red arrows) were analyzed for CD44 and RFP expression as a validation assay, using the indicated fluorescently-conjugated antibodies.

Figure S8

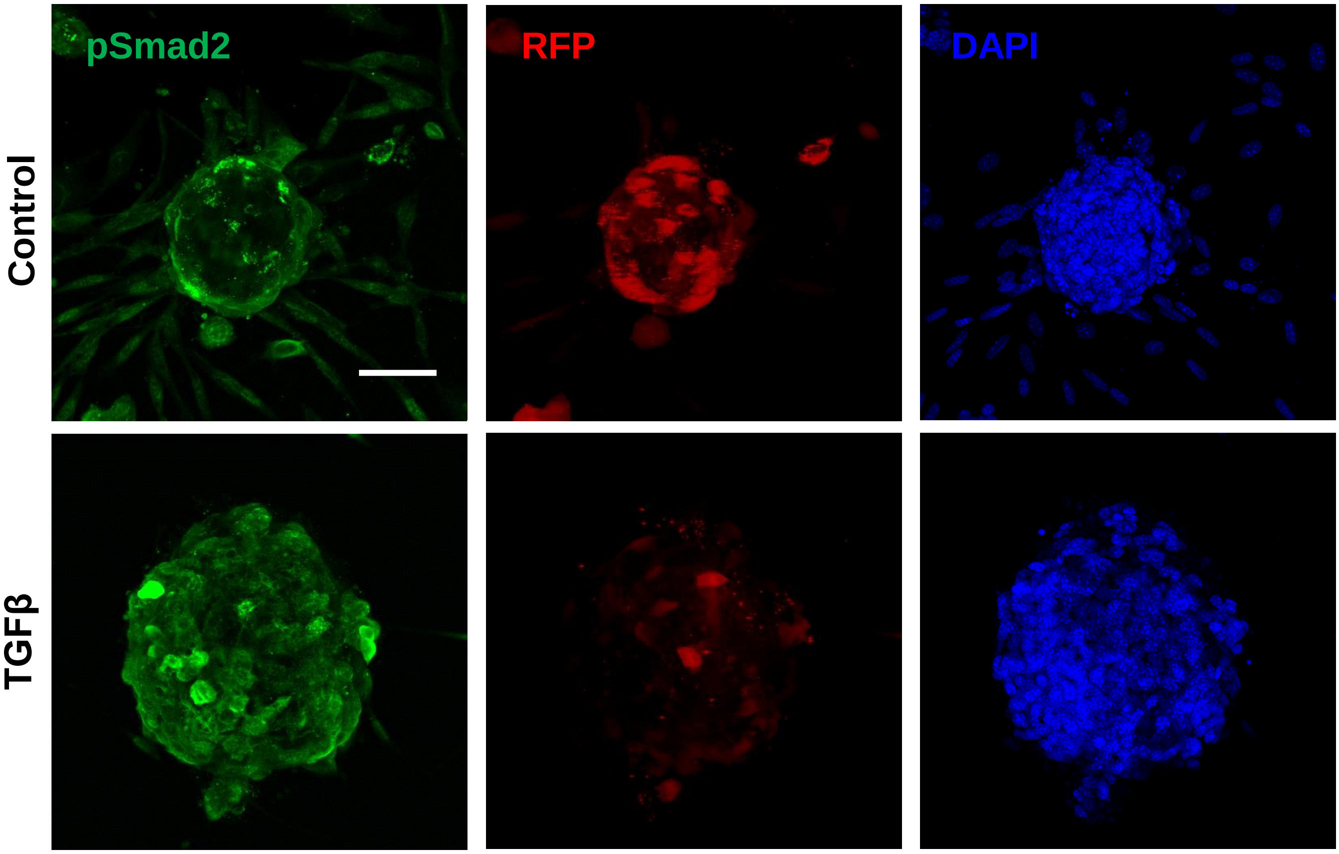

Fig. S8. Analysis of TGFβ signaling in mammospheres. (A) Representative signaling (pSmad2) in invading mammospheres (n=3 biological replicates). Representative fluorescence microscopy images for pSmad2 (green), RFP (red) and nuclei (DAPI, blue) of *E-cadherin*-RFP/Py2T mammospheres and migrating cells. Scale bar 100 μm.

Figure S9

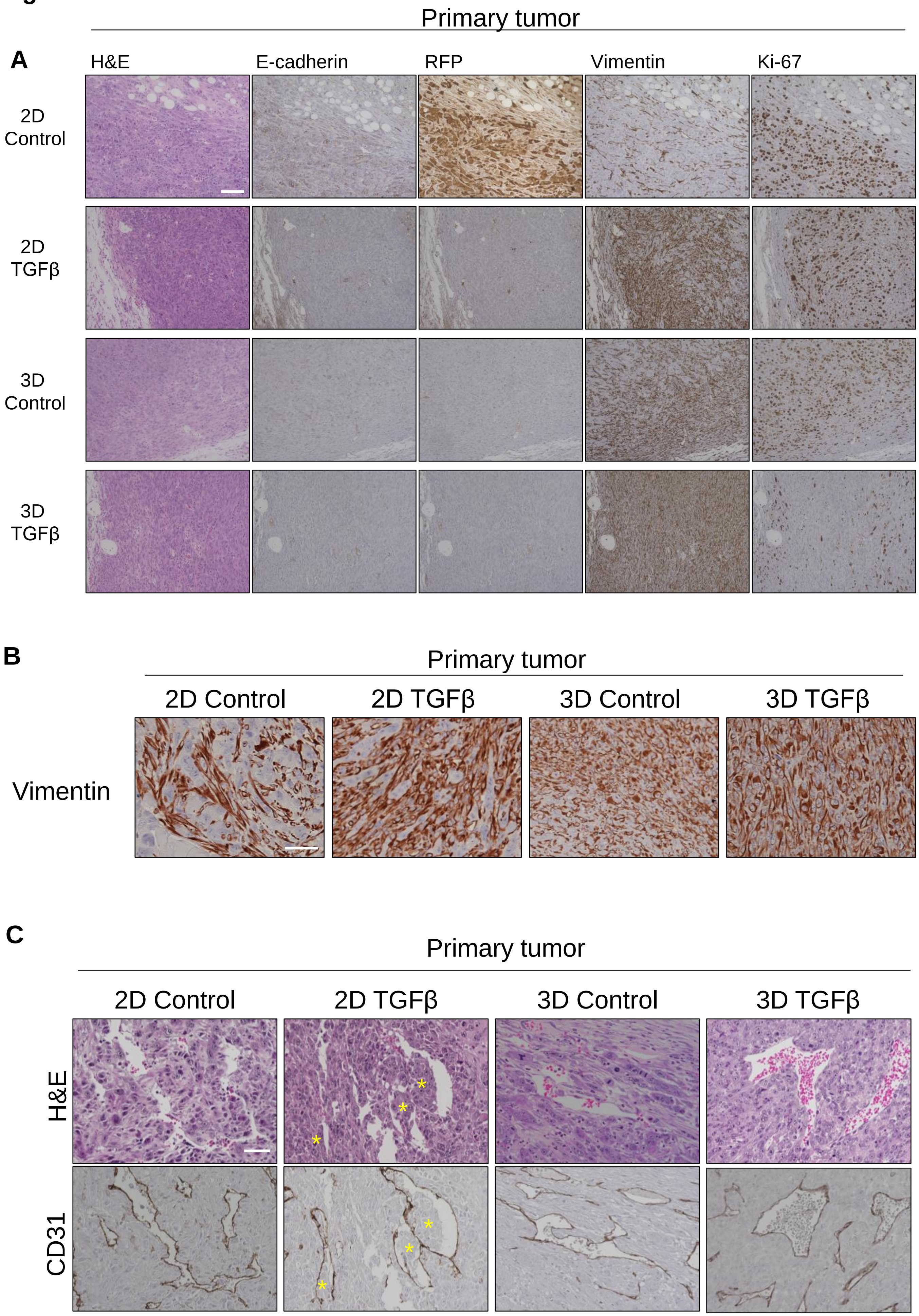
A
Primary tumor
H&E
E-cadherin
RFP
Vimentin
Ki-67
2D Control
2D TGFβ
3D Control
3D TGFβ
B
Primary tumor
2D Control
2D TGFβ
3D Control
3D TGFβ
Vimentin
C
Primary tumor
2D Control
2D TGFβ
3D Control
3D TGFβ
H&E
CD31

Fig. S9. EMTimage primary tumor analysis. (A) Representative images of primary tumors described in Fig. 7B, C, isolated and stained by H&E or the indicated antibodies after 6 weeks (n=21 independent tumors analyzed). Scale bar, 100 μm. (B) Representative images of vimentin staining in primary breast tumors generated by transplanted cells from the indicated conditions. Scale bar, 50 μm. (C) Representative images of primary breast tumor cells detected inside tumor-infiltrating blood vessels. Up, H&E staining of tumor cells and blood vessels with obvious red blood cells inside the vessel lumen. Down, anti-CD31 immunohistochemistry demarcating blood vessels within the tumor mass. Yellow stars mark tumor cell population identified in the blood vessel lumen. Scale bar, 100 μm.

Figure S10

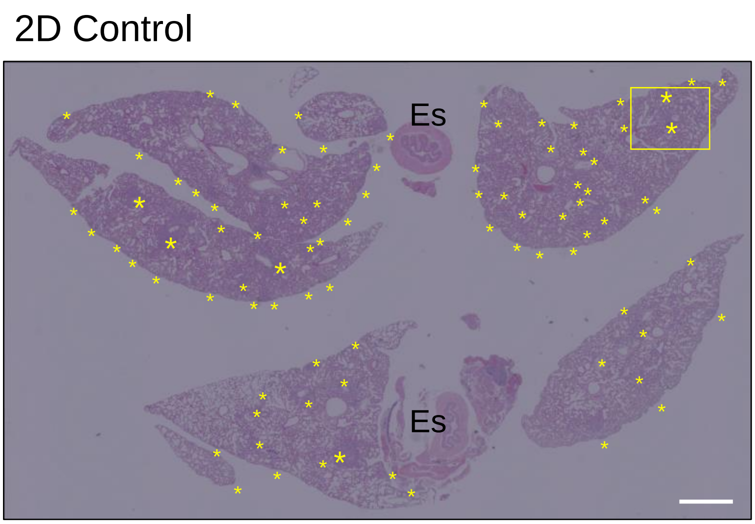

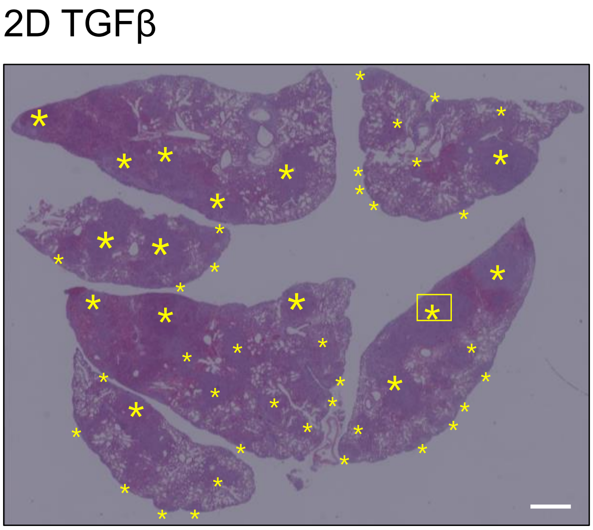

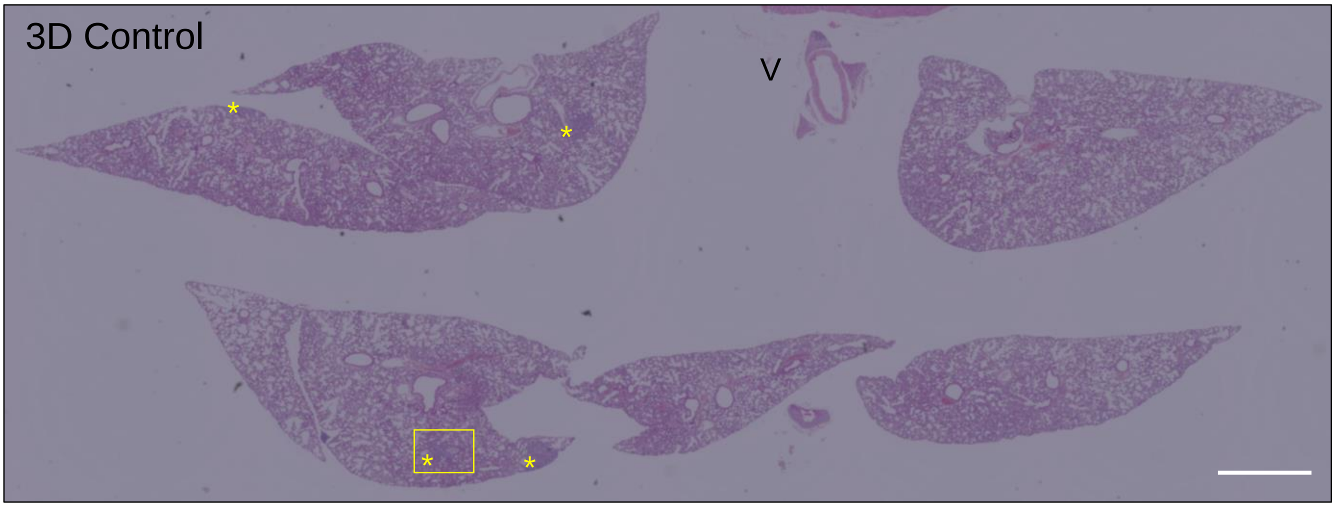

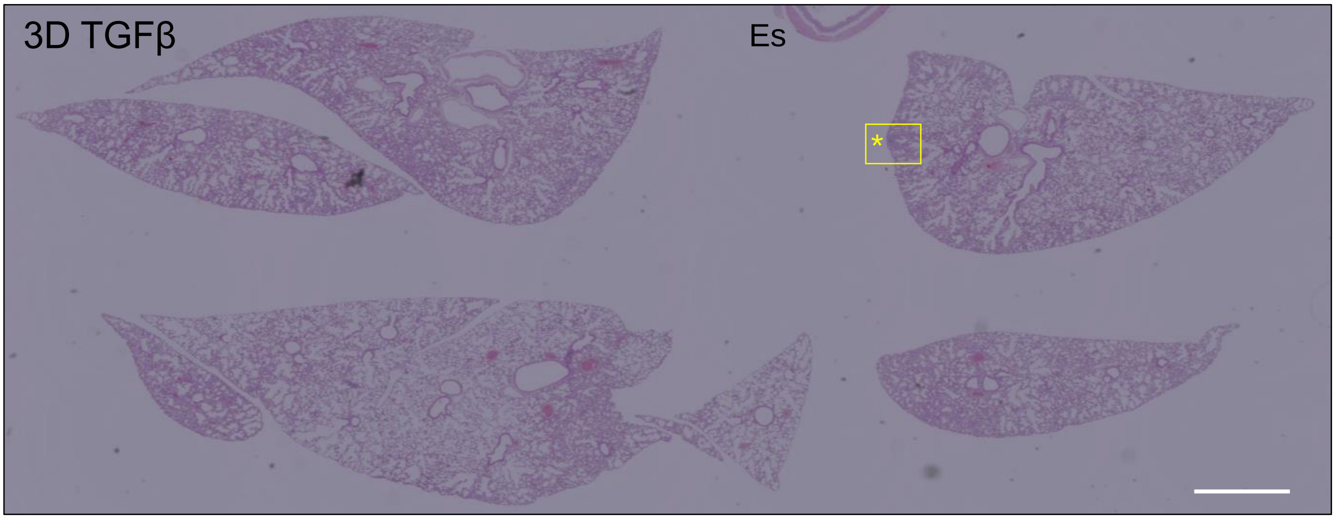

Fig. S10. EMTimage lung metastasis analysis. Representative tiling images of H&E staining. Yellow boxes demarcate the images shown in main Fig. 7D. Asterisks show metastatic nodules and asterisk size correlates to nodule size. Scale bar, 1 mm. The esophagus (Es) and a blood vessel (V) are indicated.

Figure S11

Source data Figure S1B

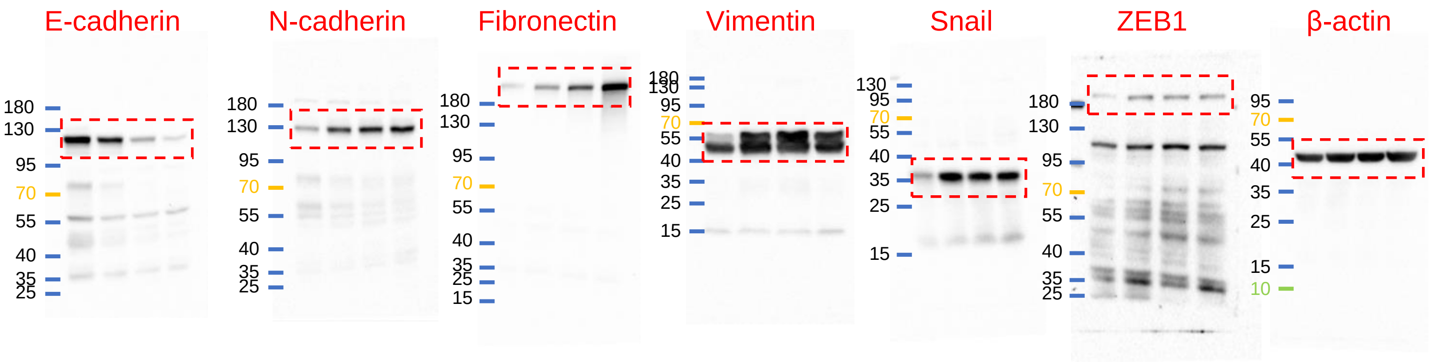

Source data Figure S1E

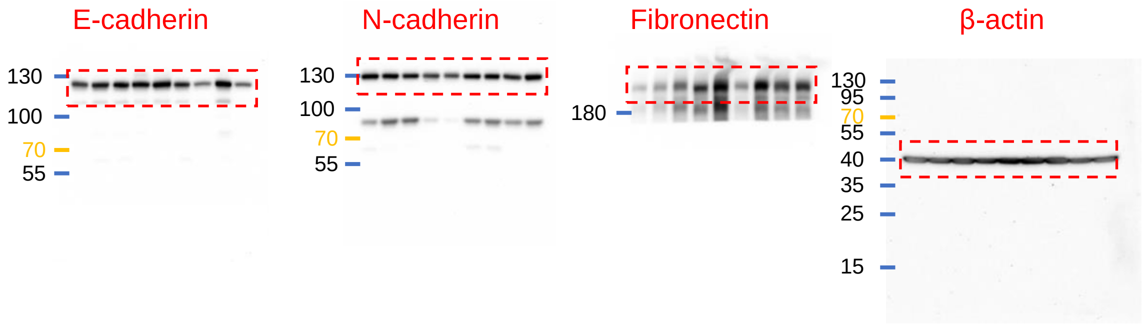

Source data Figure S1H

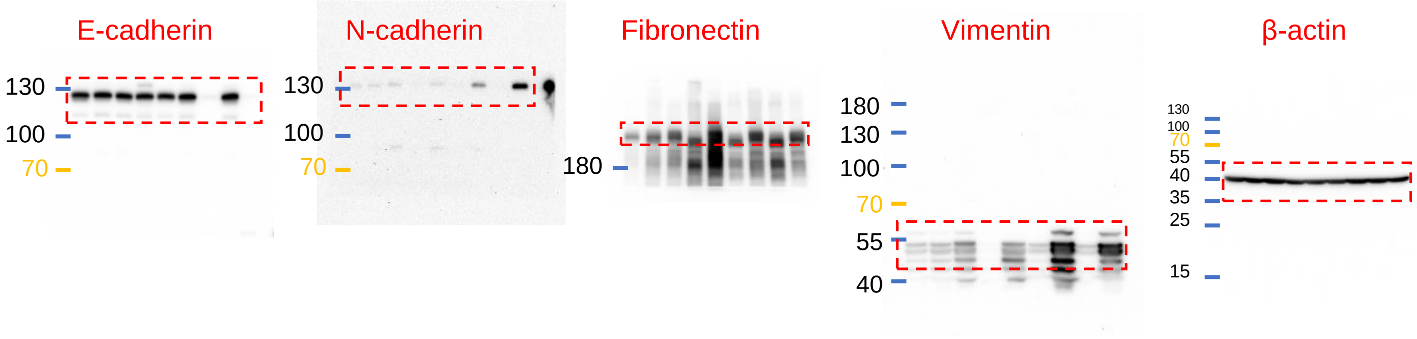

Source data Figure S1K

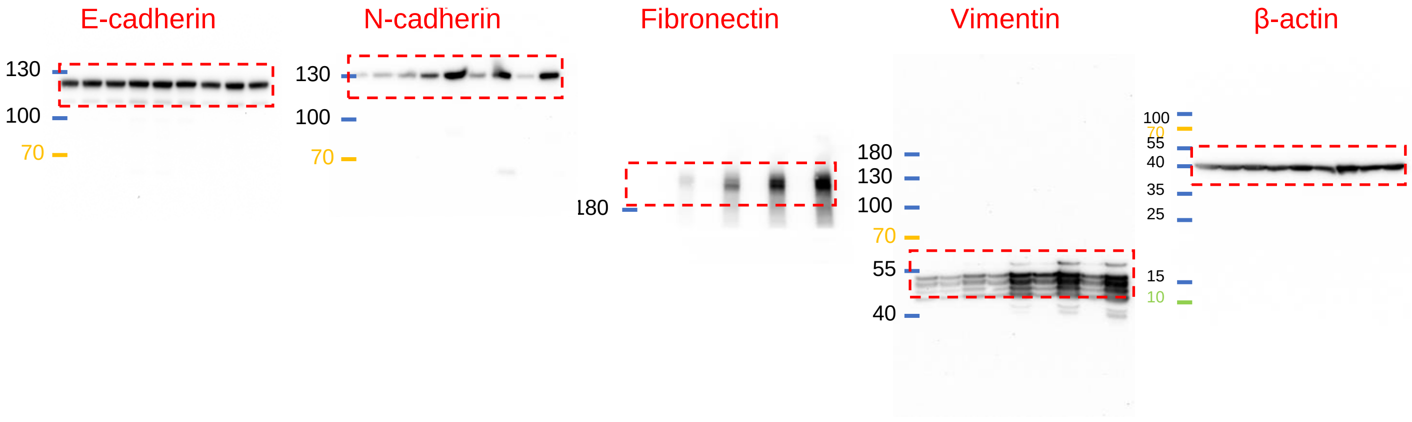

Figure S11

Source data main Figure 1C

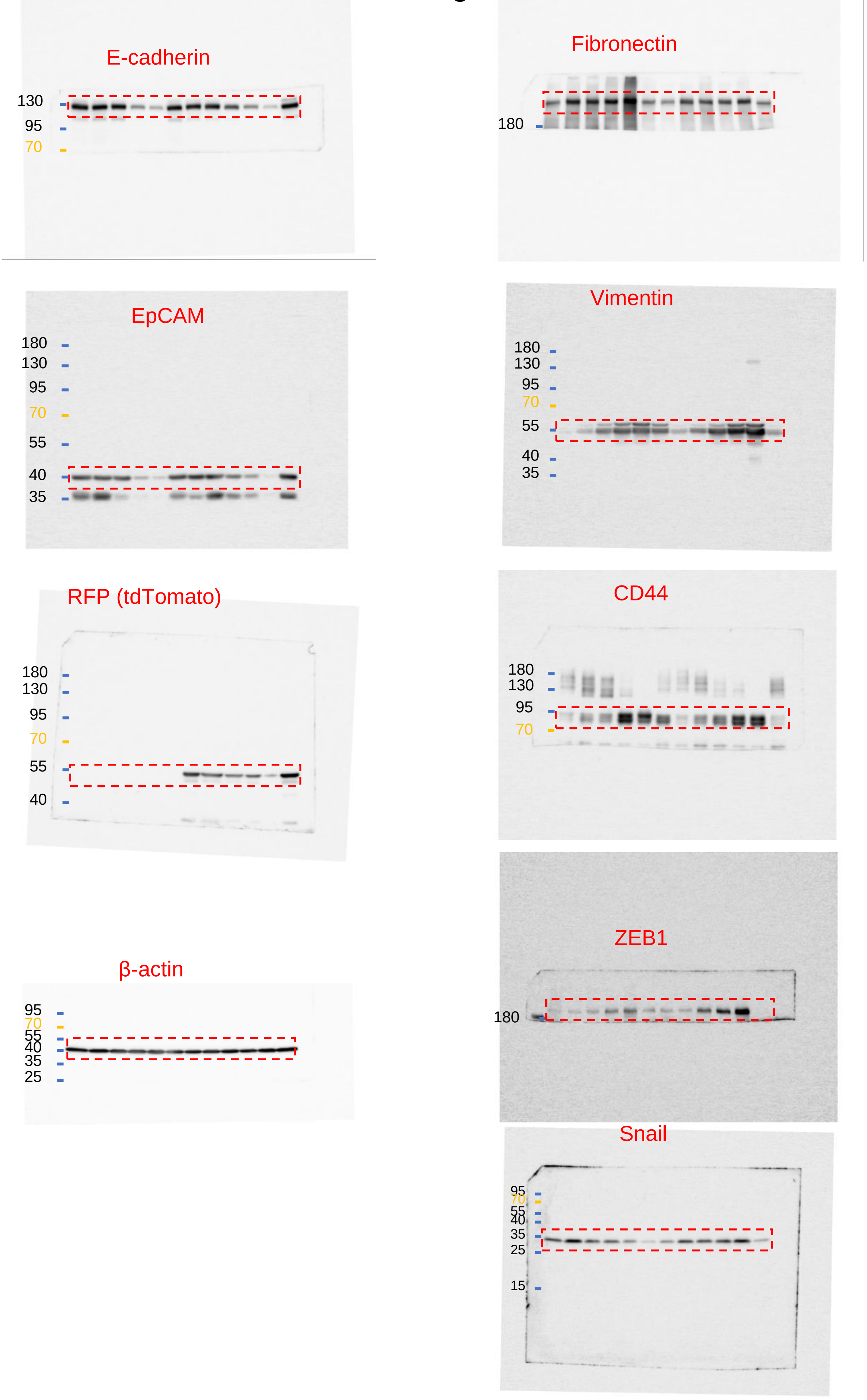

Figure S11

Source data main Figure 3D

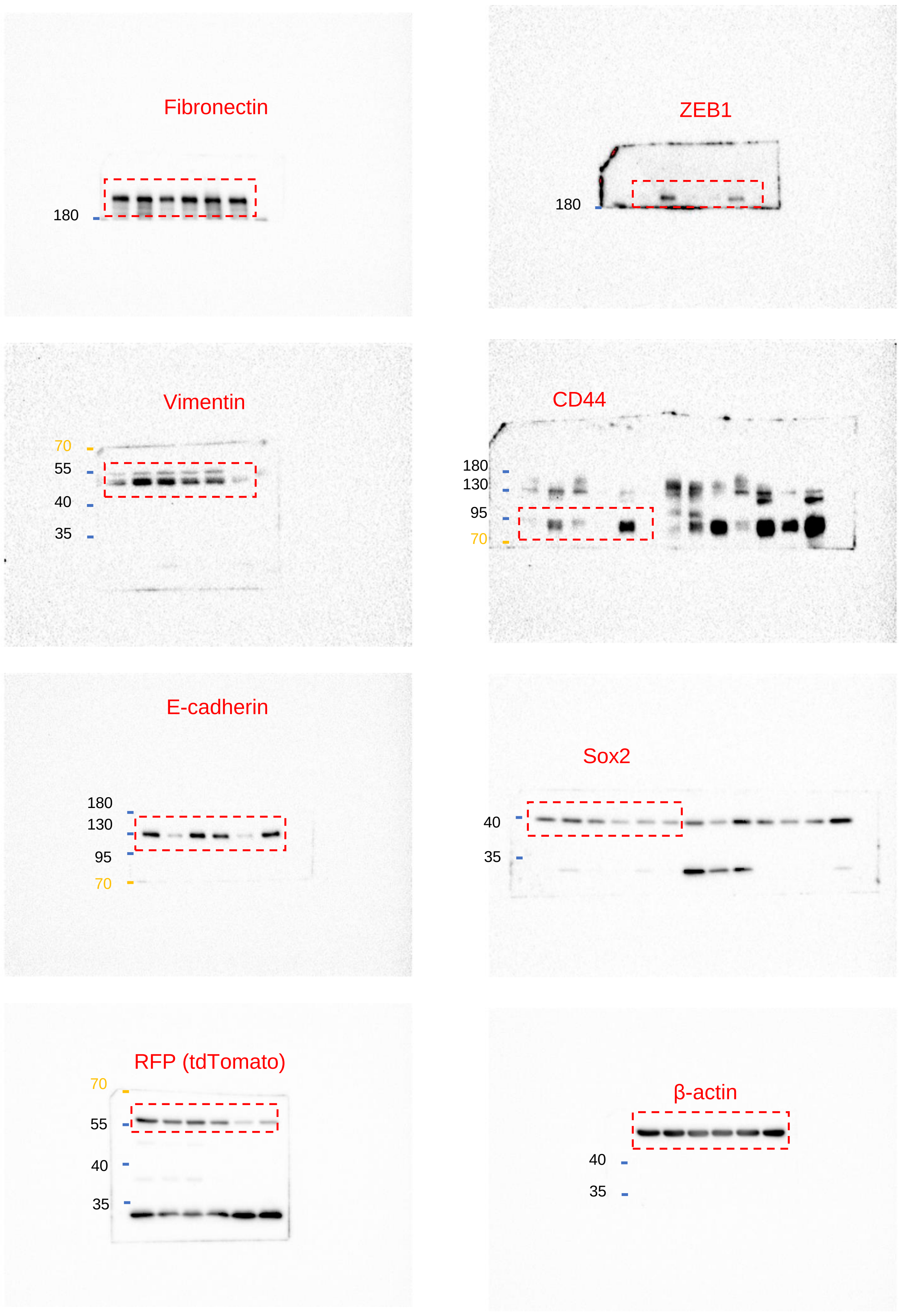

Figure S11

Source data main Figure 4D

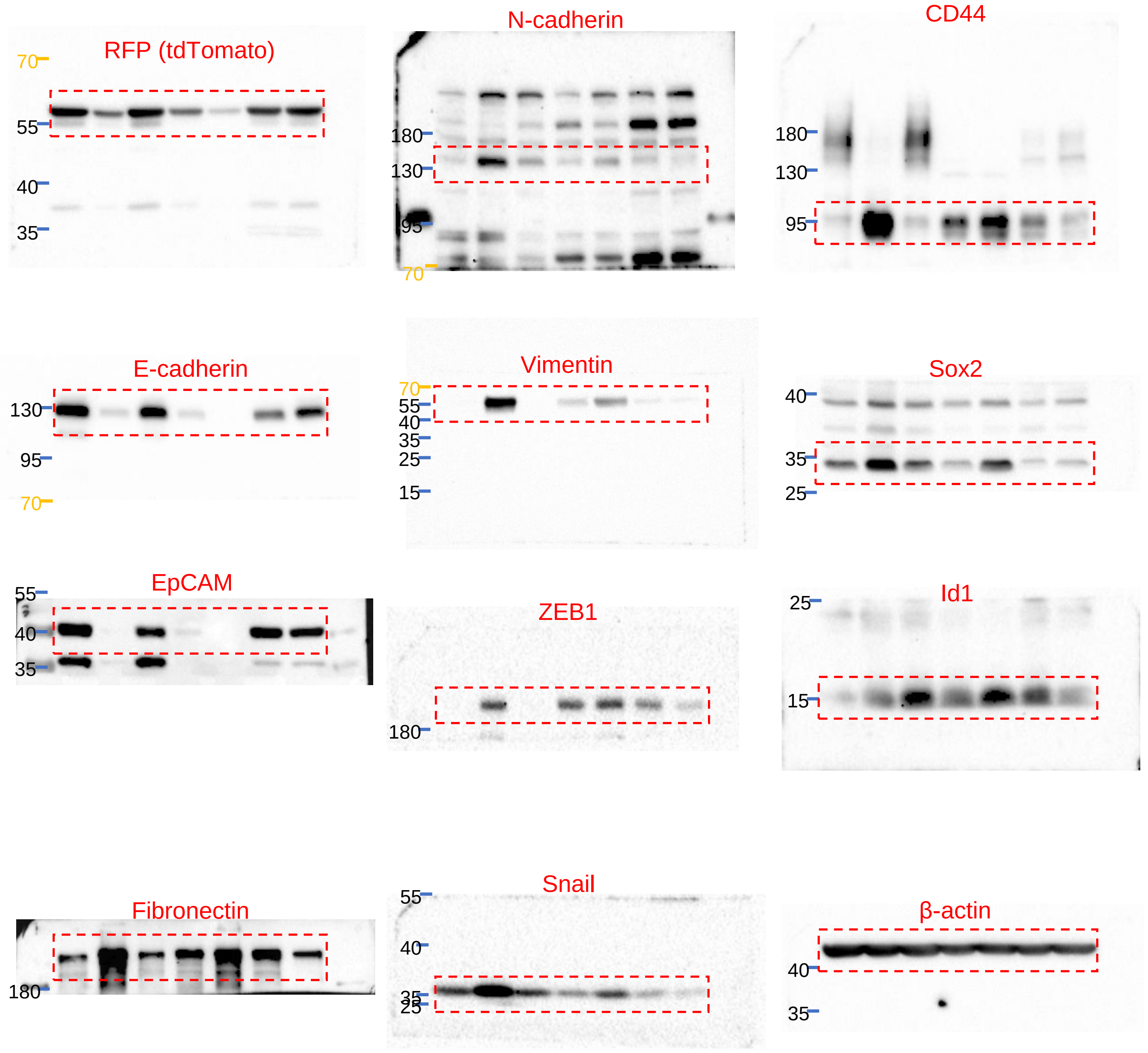

Fig. S11. Unprocessed immunoblots for the indicated proteins along with molecular size markers are shown. Dotted rectangles demarcate the cropped immunoblots presented in the main or supporting figures, as indicated in each figure panel.

Supporting movies

Movie S1. Time-lapse EMT imaging of *E-cadherin*-RFP/Py2T cells

Cells were grown and treated with 5 ng/ml TGFβ1 on a glass-bottom dish, and time-lapse imaging was performed with an TCS SP8 (Leica Microsystems, Wetzlar, Germany). Images were acquired every 15 min. Total imaging time = 48 h.

Movie S2. Time-lapse MET imaging of *E-cadherin*-RFP/Py2T cells

Seven days after stimulation with 5 ng/ml TGFβ1, cells were grown without TGFβ1 on a glass-bottom dish, and time-lapse imaging was performed with an ECLIPSE Ti2 microscope (Nikon, Minato, Japan). Images were acquired every 30 min. Total imaging time = 62 h.

Movie S3. Time-lapse imaging of *E-cadherin*-RFP/Py2T mammospheres during adhesion that elicits cell migration

Five days after mammosphere formation in ultra-low attachment condition (3D), mammospheres were grown without TGFβ (partial EMT) or with (complete EMT) on a glass-bottom dish and time-lapse imaging was performed with an ECLIPSE Ti2 microscope (Nikon, Minato, Japan). Images were acquired every 8 min. Total imaging time = 55 h.
